# Habitual intake of dietary methylglyoxal is associated with less low-grade inflammation: the Maastricht Study

**DOI:** 10.1093/ajcn/nqac195

**Published:** 2022-09-07

**Authors:** Kim Maasen, Simone J P M Eussen, Pieter C Dagnelie, Alfons J H M Houben, Carroll A B Webers, Miranda T Schram, Tos T J M Berendschot, Coen D A Stehouwer, Antoon Opperhuizen, Marleen M J van Greevenbroek, Casper G Schalkwijk

**Affiliations:** Department of Internal Medicine, CARIM School for Cardiovascular Diseases, Maastricht University Medical Center, Maastricht, The Netherlands; Department of Epidemiology, CAPHRI Care and Public Health Research Institute/CARIM School for Cardiovascular Diseases, Maastricht University Medical Center, Maastricht, The Netherlands; Department of Epidemiology, CAPHRI Care and Public Health Research Institute/CARIM School for Cardiovascular Diseases, Maastricht University Medical Center, Maastricht, The Netherlands; Department of Internal Medicine, CARIM School for Cardiovascular Diseases, Maastricht University Medical Center, Maastricht, The Netherlands; University Eye Clinic Maastricht, Maastricht University Medical Center, Maastricht, The Netherlands; Department of Internal Medicine, Heart and Vascular Center, Maastricht University Medical Center, Maastricht, The Netherlands; University Eye Clinic Maastricht, Maastricht University Medical Center, Maastricht, The Netherlands; Department of Internal Medicine, CARIM School for Cardiovascular Diseases, Maastricht University Medical Center, Maastricht, The Netherlands; Department of Pharmacology and Toxicology, NUTRIM School of Nutrition and Translational Research in Metabolism, Maastricht University Medical Center, Maastricht, The Netherlands; Office for Risk Assessment and Research, Netherlands Food and Consumer Product Safety Authority, Utrecht, The Netherlands; Department of Internal Medicine, CARIM School for Cardiovascular Diseases, Maastricht University Medical Center, Maastricht, The Netherlands; Department of Internal Medicine, CARIM School for Cardiovascular Diseases, Maastricht University Medical Center, Maastricht, The Netherlands

**Keywords:** diet, dicarbonyls, oxoaldehyde, methylglyoxal, glyoxal, 3-deoxyglucosone, observational cohort, advanced glycation endproducts, inflammation, microcirculation

## Abstract

**Background:**

Dicarbonyls are major reactive precursors of advanced glycation endproducts (AGEs). Dicarbonyls are formed endogenously and also during food processing. Circulating dicarbonyls and AGEs are associated with inflammation and microvascular complications of diabetes, but for dicarbonyls from the diet these associations are currently unknown.

**Objectives:**

We sought to examine the associations of dietary dicarbonyl intake with low-grade inflammation and microvascular function.

**Methods:**

In 2792 participants (mean ± SD age: 60 ± 8 y; 50% men; 26% type 2 diabetes) of the population-based cohort the Maastricht Study, we estimated the habitual intake of the dicarbonyls methylglyoxal (MGO), glyoxal (GO), and 3-deoxyglucosone (3-DG) by linking FFQ outcome data to our food composition database of the MGO, GO, and 3-DG content of >200 foods. Low-grade inflammation was assessed as six plasma biomarkers, which were compiled in a *z* score. Microvascular function was assessed as four plasma biomarkers, compiled in a *z*score; as diameters and flicker light–induced dilation in retinal microvessels; as heat-induced skin hyperemic response; and as urinary albumin excretion. Cross-sectional associations of dietary dicarbonyls with low-grade inflammation and microvascular function were investigated using linear regression with adjustments for age, sex, potential confounders related to cardiometabolic risk factors, and lifestyle and dietary factors.

**Results:**

Fully adjusted analyses revealed that higher intake of MGO was associated with a lower *z* score for inflammation [standardized β coefficient (STD β): −0.05; 95% CI: −0.09 to −0.01, with strongest inverse associations for hsCRP and TNF-α: both −0.05; −0.10 to −0.01]. In contrast, higher dietary MGO intake was associated with impaired retinal venular dilation after full adjustment (STD β: −0.07; 95% CI: −0.12 to −0.01), but not with the other features of microvascular function. GO and 3-DG intakes were not consistently associated with any of the outcomes.

**Conclusion:**

Higher habitual intake of MGO was associated with less low-grade inflammation. This novel, presumably beneficial, association is the first observation of an association between MGO intake and health outcomes in humans and warrants further investigation.

## Introduction

Dicarbonyls are highly reactive compounds and major precursors of advanced glycation endproducts (AGEs) (1). Dicarbonyls are formed endogenously, during glycolysis and lipid peroxidation, and they are also formed in food processing, mainly during heat treatment (2, 3). We recently showed that a higher habitual intake of the dicarbonyls MGO and GO was associated with higher concentrations of these dicarbonyls in plasma. Additionally, higher MGO intake was associated with higher autofluorescence in the skin, an estimate for AGE accumulation in tissue (4). These findings suggest that dietary dicarbonyls contribute to the presence of dicarbonyls and AGEs in the body.

In the body, dicarbonyls rapidly react with free amino acids, proteins, and DNA. This glycation reaction alters the structure and function of these compounds. MGO is thought to be the most reactive and biologically relevant dicarbonyl. MGO can be detoxified by cellular defense mechanisms, of which glyoxalase-1 is the rate-limiting enzyme for the detoxification of MGO, or MGO can be excreted into the urine (1).

Concentrations of dicarbonyls and AGEs in the circulation are associated with inflammation and microvascular dysfunction, both of which are important risk factors for microvascular complications such as retinopathy, nephropathy, and neuropathy [as reviewed in (1)]. Moreover, we previously showed that higher plasma concentrations of the major dicarbonyl MGO were associated with albuminuria, eGFR, and retinopathy (5), as well as with incident cardiovascular disease (6, 7).

It is currently unknown if, and to which extent, dicarbonyls derived from the diet also contribute to inflammation and microvascular function. Long-term exposure to high amounts of oral MGO in animals induced adverse vascular effects, such as endothelial dysfunction (8), glomerular basement membrane thickness in the kidney (9), and inflammation (8, 10). In contrast, other studies in animals showed no adverse effects after long-term MGO administration, despite elevated MGO concentrations in the circulation, and even showed an increase in antioxidant systems (11) and slightly increased survival (12). Recent experimental studies also revealed favorable effects of MGO, including antioxidative effects and prolonged lifespan (13–16). Thus, dicarbonyls intake might have both beneficial and undesirable consequences.

The consequences of dietary dicarbonyls have, to the best of our knowledge, never been studied in humans. Therefore, in the current study, performed in a large population-based cohort, we examined the associations of dietary dicarbonyls with plasma biomarkers of low-grade inflammation and with markers of microvascular function, defined as biomarkers of endothelial function in plasma, as diameter and flicker light-induced dilation in the venules and arterioles of the retina, as heat-induced hyperemia in the skin, and as urinary albumin excretion in the kidney.

## Methods

### Study population

This study used data from the Maastricht Study, an observational prospective population-based cohort study. The rationale and methodology have been described previously (17). In brief, the study focused on the etiology, pathophysiology, complications, and comorbid conditions of type 2 diabetes and was characterized by an extensive phenotyping approach. Study patients eligible for participation were all individuals aged 40 to 75 y and living in the southern part of the Netherlands. Participants were recruited through mass media campaigns and from municipal registries and the regional Diabetes Patient Registry by mailings. Recruitment was stratified according to known type 2 diabetes status, with an oversampling of individuals with type 2 diabetes for reasons of efficiency. The present report includes cross-sectional data from the first 3451 participants, who completed the baseline survey between November 2010 and September 2013. Follow-up information on the outcomes of interest in this study is not yet available. The examinations of each participant were performed within a time window of 3 mo. The study was approved by the institutional medical ethics committee (NL31329.068.10) and the Minister of Health, Welfare and Sports of the Netherlands (permit 131,088–105,234-PG) and was conducted in accordance with the Declaration of Helsinki. All participants gave written informed consent.

### Assessment of dietary MGO, GO, and 3-DG intake

Habitual intake of the dicarbonyls MGO, GO, and 3-DG was estimated as previously described (4). In brief, we combined food intake data from the FFQ used in The Maastricht Study (18) with our previously published dietary dicarbonyl database, containing MGO, GO, and 3-DG concentrations of 223 foods and drinks (19).

To estimate habitual daily dietary intake of MGO, GO, and 3-DG, we multiplied the dicarbonyl concentration of a food product (mg/g) by the individual's estimated daily intake of that food product based on the FFQ (grams per day), and subsequently summed all 253 food items from the FFQ (see formula below, using MGO as example).

(1) \begin{eqnarray*} && MGO\,\,intake\,(mg/day)\\ &&\quad = \sum\nolimits_{i = 1}^n {([MGO]i\,\,(mg/g) * intake}\,\,i\,\,(g/day)) \end{eqnarray*}

Where *i* is the food item, [*MGO*]*i* is MGO concentration for that particular food item from the database, and intake *i* is intake of that particular food item derived from the FFQ.

### Assessment of low-grade inflammation

Generalized low-grade inflammation can be assessed as higher concentrations of proinflammatory cytokines in plasma. Plasma samples were collected after an overnight fast and stored at −80°C until measurements. We measured 6 plasma biomarkers of low-grade inflammation: high sensitivity C-reactive protein (hsCRP), serum amyloid A (SAA), soluble intercellular adhesion molecule-1 (sICAM-1), IL-6, IL-8, and tumor necrosis factor alpha (TNF-α). These biomarkers were measured in EDTA plasma samples with commercially available 4-plex sandwich immunoassay kits [Meso Scale Discovery (MSD)], as reported before (20).

### Assessment of microvascular function

Microvascular function can be assessed noninvasively in various organs. In the eye, it can be assessed as wider or narrower retinal arteriolar and venular diameters, or impaired flicker light–induced retinal arteriolar and venular dilation response. For retinal venular diameter, microvascular dysfunction is reflected by widening, whereas for retinal arteriolar diameter this is less clear, but it is thought that the development of diabetic microvascular dysfunction is reflected by initial widening and later narrowing (21). In the skin, it can be assessed as impaired heat-induced skin hyperemia (22). Microvascular function can also be assessed as higher urinary albumin excretion, a measure of kidney microvascular dysfunction (21), and as higher plasma biomarkers of endothelial function (23).

For the retinal and skin measurements, all participants were asked to refrain from smoking and drinking caffeine-containing beverages 3 h before the measurement. A light meal (breakfast or lunch), low in fat content, was allowed at least 90 min before the start of the measurements (22).

#### Plasma biomarkers of endothelial function

Four plasma biomarkers of endothelial function were measured: sICAM-1, sVCAM-1, soluble E-selectin (sE-selectin), and von Willebrand factor (vWF). sICAM-1, sVCAM-1, and sE-selectin were measured in EDTA plasma samples with commercially available 4-plex sandwich immunoassay kits with different standards and antibodies (Meso Scale Discovery). vWF was quantified in citrate plasma using ELISA (Dako). Concentrations of vWF were expressed as a percentage of vWF detected in pooled citrated plasma of healthy volunteers (20, 22).

#### Retinal microvascular diameters

All fundus photographs were taken with an autofocus, autoshot, and autotracker fundus camera (Model AFC-230, Nidek) in an optic disc–centered field of view of 45° in a darkened room, as described previously (22). Static retinal vessel analysis (one image of the left or right eye, randomly chosen) was performed with the RHINO software (Eindhoven). Optic disc detection and arteriole/venule classification were corrected manually. Retinal vessel diameters were measured 0.5–1.0 disc diameters away from the optic disc margin and were presented as central retinal arteriolar and venular equivalent (CRAE and CRVE, respectively) in measurement units (MU). The scale factor is based on the optic disc diameter, which is assumed to be 1800 μm. CRAE and CRVE represent the equivalent single-vessel parent diameter for the 6 largest arterioles and largest venules in the region of interest, respectively. The calculations were based on the improved Knudston–Parr–Hubbard formula (24). Fundus photographs of insufficient quality, e.g., obstructed by lashes or defocused, were evaluated and discussed with a second observer and excluded on mutual agreement.

#### Flicker light–induced retinal microvascular dilation response

The retinal arteriolar and venular dilation response to flicker-light exposure was assessed by the Dynamic Vessel Analyzer (DVA; Imedos, Jena), as previously described (22). Briefly, pupils were dilated with 0.5% tropicamide and 2.5% phenylephrine at least 15 min prior to the start of the examination. For safety reasons, participants with an intraocular pressure >30 mm Hg were excluded from the measurements. Per participant, either the left or the right eye was selected depending on the time of day the measurement was performed and without reference to participant characteristics. A straight arteriolar or venular segment of ∼1.5 mm in length located 0.5–2 disc diameters from the margin of the optic disc in the temporal section was examined. Vessel diameter was automatically and continuously measured for 150 s. A baseline recording of 50 s was followed by a 40-s flicker light exposure period (flicker frequency 12.5 Hz, bright-to-dark contrast ratio of 25 to 1), followed by a 60-s recovery period. Baseline retinal vascular diameters and flicker light–induced retinal vascular dilation were automatically calculated with the integrated DVA software (version 4.51, Imedos). Baseline retinal arteriolar/venular diameter was calculated as the average diameter of the 20- to–50-s recording and was expressed in measurement units, where one measurement unit is equal to 1 mm of the Gullstrand eye.

The flicker light–induced retinal vascular dilation was expressed as the percentage retinal vascular dilation over baseline and based on the average dilation achieved at time points 10 and 40 s during the flicker stimulation period. This dilation response depends on a process called neurovascular coupling, which involves endothelial function (22, 25).

#### Heat-induced skin hyperemic response

Skin blood flow was measured as described previously by means of a laser-Doppler system (Periflux 5000, Perimed) equipped with a thermostatic laser-Doppler probe (PF457, Perimed) at the dorsal side of the wrist of the left hand (22). The laser-Doppler output was recorded for 25 min with a sample rate of 32 Hz, which gives semiquantitative assessment of skin blood flow expressed in arbitrary perfusion units (PU). Skin blood flow was first recorded unheated for 2 min to serve as a baseline. After the 2 min of baseline, the temperature of the probe was rapidly and locally increased to 44°C and was then kept constant until the end of the registration. The heat-induced skin hyperemic response was expressed as the percentage increase in average perfusion units during the 23-min heating phase over the average baseline perfusion units. Skin perfusion during a period of local heating is thought to be mainly endothelium dependent (26, 27), and this method is commonly used as a test of skin microvascular function (28).

#### Urinary albumin excretion

Two 24-h urine collections were used to assess urinary albumin excretion. Urinary albumin concentration was measured with a standard immunoturbidimetric assay by an automatic analyzer (due to a change of supplier by the Beckman Synchron LX20, Beckman Coulter Inc., and the Roche Cobas 6000 F, Hoffmann-La Roche) and multiplied by collection volume to obtain the 24 h urinary albumin excretion (22). A urinary albumin concentration below the detection limit of the assay (2 mg/l for the Beckman Synchron LX20 and 3 mg/l for the Roche Cobas 6000) was set at 1.5 mg/l before multiplying by collection volume. Only urine collections with a collection time between 20 and 28 h were considered valid. If needed, urinary albumin excretion was extrapolated to a 24-h excretion. Increased urinary albumin excretion (i.e., albuminuria) is as a risk marker for generalized endothelial dysfunction (29).

### Assessment of other covariates

Glucose metabolism status was assessed by a 75-g oral glucose tolerance test (OGTT) and defined according to WHO 2006 criteria as normal glucose metabolism, prediabetes [impaired fasting glucose (6.1–7.0 mmol/L) and/or impaired glucose tolerance (2-h glucose 7.8–11.1 mmol/L)], type 2 diabetes (fasting plasma glucose ≥7.1, 2-h glucose >11.1, or the use of diabetes medication) or other types of diabetes.

Weight and height were measured by a trained staff member, and BMI was was measured in kg/m^2^. Age, sex, smoking behavior, history of cardiovascular disease, educational level, and presence of gastrointestinal tract infection (defined as self-reported symptoms of gastrointestinal tract infection in the previous 2 mo) were assessed by means of a self‐report questionnaire (17). Physical activity was assessed using the CHAMPS questionnaire (30). Medication use was assessed by interview (17). Estimated glomerular filtration rate (eGFR) was calculated with the Chronic Kidney Disease Epidemiology Collaboration (CDK-epi) equation, using both serum creatinine and serum cystatin C (31). Intake of energy, macronutrients, and the Dutch Healthy Diet index, a measure of diet quality, were assessed using the FFQ described above. The Dutch Healthy Diet index used in the Maastricht Study consists of all 15 components except coffee, because the coffee component is based on filtered compared with unfiltered coffee, and our FFQ does not distinguish between these types of coffee. Hence, the Dutch Healthy Diet index score ranges from 0 (no adherence) to 140 (complete adherence) (32). Office and 24-h ambulatory blood pressure, total cholesterol, HDL cholesterol, triglycerides, fasting plasma glucose, and glycosylated hemoglobin (HbA1c) were determined as described elsewhere (17).

### Statistical analyses

The general characteristics of the study population are shown for the total sample and compared across tertiles of total dietary dicarbonyl intake. For this, a standardized composite score of total dietary dicarbonyl intake was calculated by standardizing each dietary dicarbonyl (MGO, GO, and 3-DG), then averaging these results into an overall dietary dicarbonyl *z*-score. Differences in characteristics among individuals in the tertiles were tested using one-way ANOVA for normally distributed continuous variables, the Kruskal–Wallis test for nonnormally distributed continuous variables, and the chi-square test for discrete variables. We also evaluated to what extent participants who were excluded from the analyses due to missing covariates differed from those who were included.

For all further analyses, each dietary dicarbonyl was considered individually, since they are thought to exert different biological effects (33). Standardized composite scores were created for plasma biomarkers of low-grade inflammation and endothelial function, for reasons of statistical efficiency and to reduce the influence of the biological variability of each biomarker. Before calculating the composite scores, all nonnormally distributed markers (i.e., hsCRP, SAA, sICAM-1, IL-6, IL-8, TNF-α, sE-selectin) were ln-transformed. The standardized composite scores were calculated by standardizing each individual biomarker, then averaging these results into overall standardized composite scores for either low-grade inflammation or endothelial function, and then standardized again. The low-grade inflammation composite score consisted of the biomarkers hsCRP, SAA, sICAM-1, IL-6, IL-8, and TNF-α, and the endothelial function composite score consisted of the biomarkers sVCAM-1, sICAM-1, sE-selectin, and vWF.

For the other outcome variables, urinary albumin excretion was ln-transformed to obtain a normal distribution. Absolute change over baseline (delta) retinal venular dilation was used as the outcome in the association with MGO intake, because baseline retinal venular dilation was significantly associated with MGO intake (**Supplemental Table S1**) and spurious associations between determinants and outcomes expressed as percentage may occur when determinants are associated with baseline measures (34). Perfusion during the 23-min heating phase (in PU) was used as outcome in the association with 3-DG intake, because baseline skin blood flow was also significantly associated with 3-DG intake (Supplemental Table S1). Analyses in which absolute perfusion was used as the outcome were additionally adjusted for baseline skin blood flow in all regression models [this will not lead to autocorrelation because there was only a weak association between baseline skin blood flow and heat-induced skin hyperemia (34)]. All outcome variables and main independent variables were standardized to allow direct comparison of the strength of the associations.

We examined the associations of each standardized dietary dicarbonyl (MGO, GO, and 3-DG) with standardized low-grade inflammation and with standardized features of microvascular function, using multiple linear regression analyses. Associations were first adjusted for age (years), sex (males/females), glucose metabolism status (prediabetes, type 2 diabetes, or other types of diabetes as dummy variables with normal glucose metabolism as reference category), and baseline skin blood flow (only for analyses of heat-induced skin hyperemia) (model 1). Next, additional adjustments were made for potential confounders related to lifestyle, i.e., BMI, smoking (“former” or “current” as dummy variables with “never” as reference category), alcohol intake (grams/day), physical activity (total score of all activities, hours/week), total energy intake (kcal/day), and education (“medium” or “high” as dummy variables with “low” as the reference category) (model 2). Finally, the analyses were additionally adjusted for other cardiovascular risk factors: triglycerides (mmol/L), systolic blood pressure (mm Hg), total cholesterol/HDL ratio, use of glucose-lowering-, antihypertensive, or lipid-modifying drugs (yes/no) (model 3). Variables were selected as potential confounders if they were known, or expected, to be associated with both the predictor and the outcome based on biological knowledge and literature.

Because the associations between intake of dietary components and (health) outcomes are often nonlinear (35), we tested for deviation from a linear trend in our associations. For this test, regression models using dietary dicarbonyls as continuous and categorical exposures were compared with a likelihood ratio test. None of the models showed a significantly better fit of the data when using dietary dicarbonyls as categorical exposures, and therefore dietary dicarbonyls were entered as continuous exposures in all models.

We did not adjust for multiple testing because this would increase the chance that a real biological association would remain undetected (type 2 error), something that is undesirable in a hypothesis-driven explorative study (36). For each outcome measure we tested interaction terms with sex, glucose metabolism status (prediabetes and type 2 diabetes as dummy variables with normal glucose metabolism status as reference), and eGFR (mL/min/1.73 m^2^, thereby also adding eGFR as a covariate in the fully adjusted model), to evaluate whether the associations differed according to these factors. Interaction with eGFR was tested because dicarbonyls are excreted into the urine via the kidney and hence dietary dicarbonyls might have more pronounced effects in individuals with impaired kidney function. Analyses for which we found an interaction (*P* < 0.1) were stratified for sex, glucose metabolism status (normal glucose metabolism status, prediabetes, or type 2 diabetes), or eGFR (<60 and ≥60 mL/min per 1.73 m²).

In additional analyses, we explored whether observed associations were driven by any of the main food sources of dicarbonyls. For this, we adjusted model 3 sequentially for dicarbonyl intake via each of the main food groups that contributed to ≥5% of the total daily dicarbonyl intake in this population (e.g., additional adjustment for MGO intake from coffee) (4).

Several sensitivity analyses were performed to assess the robustness of the outcomes of the main analyses. First, to examine whether the associations could be attributed to intake of other dietary components or better adherence to the Dutch Healthy Diet Index 2015 (DHD-15), the fully adjusted model was, on top of energy intake (kcal/day), additionally adjusted for either carbohydrate intake, fat intake, protein intake (grams/day), or the DHD-15. In the model where we adjusted for the DHD-15, we did not separately adjust for alcohol intake, which is included in the index. Second, we additionally adjusted the fully adjusted models for eGFR (mL/min/1.73 m^2^), urinary albumin excretion (mg/24 h, except for analyses in which urinary albumin excretion was the outcome), retinopathy (yes/no), and history of cardiovascular diseases (yes/no). These covariates may introduce overadjustment, as they also reflect microvascular function. Third, the analyses were repeated after exclusion of individuals with other types of diabetes, after exclusion of individuals with current infection (defined as CRP >10 μg/mL, only for analyses with low-grade inflammation as outcome), and after exclusion of individuals with self-reported gastrointestinal tract infection. Fourth, the analyses were repeated after exclusion of individuals with previously diagnosed type 2 diabetes, as these individuals might have adapted their dietary behavior or might be more prone to underreport their food intakes. Fifth, we explored possible confounding by antihypertensive medication through further specification into RAAS inhibitors and other types of antihypertensive medication. Sixth, several covariates were substituted by alternative measurement methods. Glucose metabolism status was substituted for HbA1c, fasting plasma glucose, or postload glucose. Office blood pressure was substituted for blood pressure obtained during 24-h ambulatory measurements. Educational level was substituted for equivalent income or occupational status. Physical activity obtained from the CHAMPS questionnaire was substituted by accelerometer data (ActivPAL). We did not include accelerometer-assessed physical activity in the main analyses because data were missing for a relatively large number of participants. BMI was substituted by waist circumference.

In addition, the association between dietary dicarbonyl intake and low-grade inflammation was verified in the Cohort on Diabetes and Atherosclerosis Maastricht (CODAM), since dietary intake data and plasma biomarkers of low-grade inflammation were available in this cohort (37). CODAM included 574 individuals with a moderately increased risk for type 2 diabetes and cardiovascular disease, who were extensively characterized at baseline between 1999 and 2002. Baseline data of 515 individuals were included in these analyses (see flowchart in **Supplemental Figure S1**). We examined the cross-sectional associations of each dietary dicarbonyl (MGO, GO, and 3-DG) with low-grade inflammation using multiple linear regression, with adjustment for the same covariates as in the Maastricht Study, except for education, which was not available in this cohort.

We report β coefficients with their 95% CIs, representing the SD difference in standardized outcome per 1 SD higher intake of MGO, GO, or 3-DG per day. Statistical significance was set at *P* < 0.05, except for testing for interaction, for which statistical significance was set at *P* < 0.10. All analyses were performed using the Statistical Package for Social Sciences (SPSS, version 26.0).

## Results

### Characteristics of the study population

**FIGURE 1:**
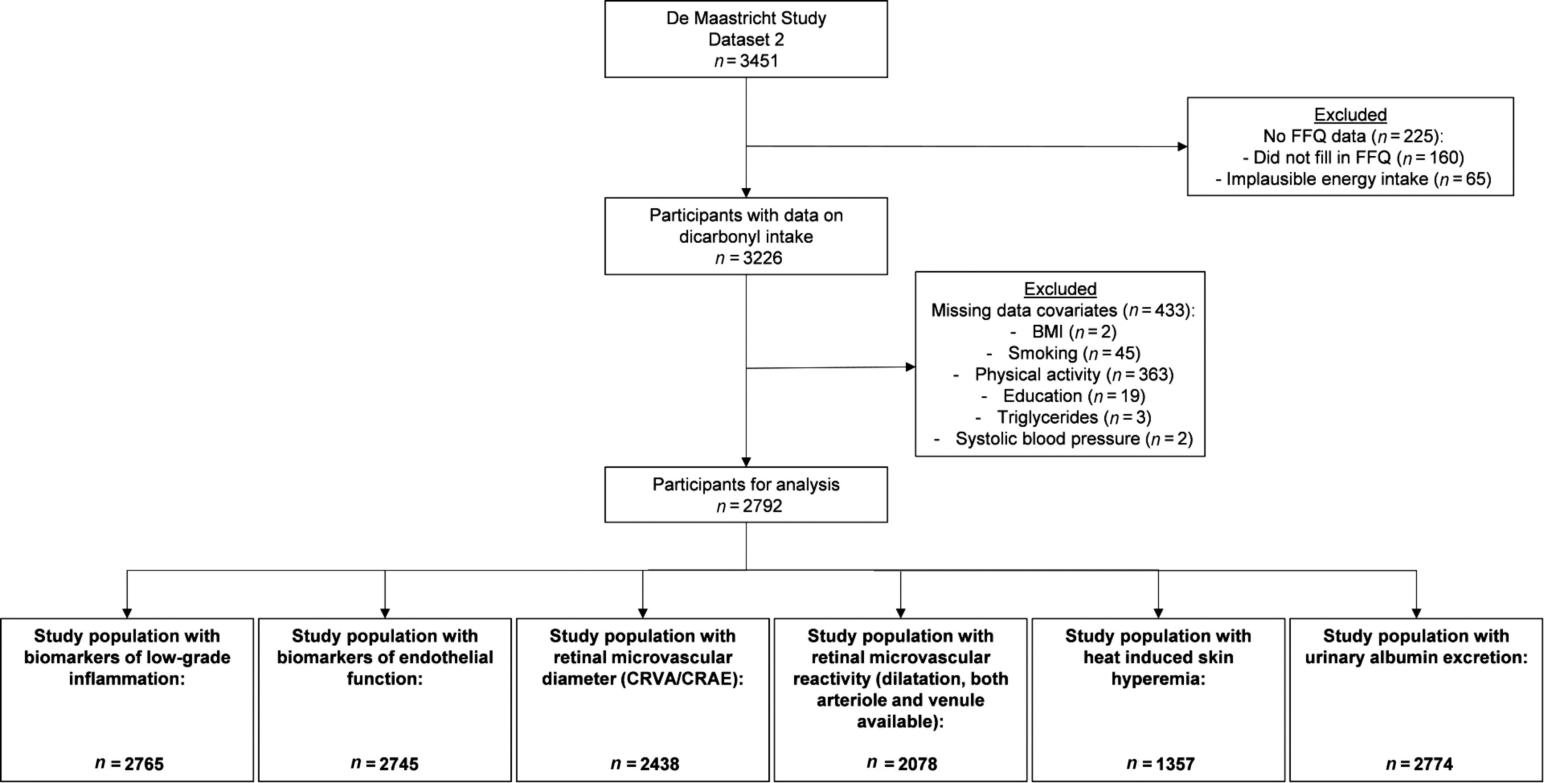
Flowchart of study population. Arteriolar dilation available for *n* = 2104 and venular dilation available for *n* = 2147. All measurements were performed at baseline. The examinations of each participant were performed within a time window of 3 mo, except for the retinal assessment, for which in a small proportion of individuals data were collected during catch-up visits. The 6 biomarkers of low-grade inflammation were hsCRP, SAA, sICAM-1, IL-6, IL-8, and TNF-α and were available in all participants (*n* = 2765) of the indicated subset. The 4 biomarkers of endothelial function were sICAM-1, sVCAM-1, sE-selectin, and vWF and were available in all participants (*n* = 2745) of the indicated subset. CRAE, central retinal arteriolar equivalent; CRVE, central retina venular equivalent; hsCRP, high sensitivity C-reactive protein; SAA, serum amyloid A; sE-selectin, soluble E-selectin; sICAM-1, soluble intercellular adhesion molecule-1; vWF, and von Willebrand factor.

Of the 3451 participants, 225 were excluded because they did not complete the FFQ (*n* = 160) or reported implausible energy intake, i.e., <800 or >4200 kcal/d for males and <500 or >3500 kcal/d for females (*n* = 65) (38) (Figure 1). Another 434 individuals were excluded because of missing data on one or more of the outcome variables or confounders. Of the remaining 2792 participants, plasma biomarkers of low-grade inflammation, plasma biomarkers of endothelial function, retinal microvascular diameters, flicker light–induced retinal microvascular dilation, heat-induced skin hyperemia, and urinary albumin excretion data were available in subpopulations of 2765, 2745, 2438, 2078, 1357, and 2774 participants, respectively. These subpopulations were comparable with regard to age, sex, and cardiovascular risk profile (**Supplemental Table S2**). Individuals with missing data on any of the covariates were more often men, and had somewhat higher cardiovascular risk factors, including more often type 2 diabetes, more current smokers, lower education, higher fasting plasma glucose, a higher total-to–HDL cholesterol ratio, and more medication use (Supplemental Table S2).

Median [IQR] dietary intake was 4.0 [3.2–4.8] mg/d for MGO, 3.5 [2.9–4.3] mg/d for GO, and 17 [12–23] mg/d for 3-DG (Table 1). Individuals in the highest tertile of overall dietary dicarbonyl intake were more often men, less often had type 2 diabetes, were more physically active, less often used glucose-lowering medication, had higher intake of energy and all other dietary components, but did not differ in adherence to the Dutch dietary guidelines. These participants also had lower plasma hsCRP, SAA, and IL-8 concentrations.

**TABLE 1:** Population characteristics for individuals with information on all confounders^1^

		Dietary dicarbonyl intake (tertiles)^2^			
Characteristics	Total population ( *n* = 2792)	Lowest (*n* = 930)	Middle (*n* = 931)	Highest (*n* = 931)	*P* value
Demographics					
Age, y	60 ± 8	60 ± 8	60 ± 8	60 ± 8	0.50
Sex, % male	50	42	51	58	<0.001
Dietary intake					
Energy intake, kcal/d	2184 ± 602	1717 ± 381	2167 ± 416	2668 ± 561	<0.001
Carbohydrate, total, g/d	233 ± 70	175 ± 40	229 ± 43	296 ± 62	<0.001
Fat, total, g/d	84 ± 31	68 ± 24	85 ± 26	101 ± 32	<0.001
Protein, g/d	86 ± 23	72 ± 17	85 ± 18	101 ± 23	<0.001
Fiber, g/d	27 ± 8.1	21 ± 5.0	27 ± 5.4	34 ± 7.8	<0.001
Alcohol intake, g/d	8.5 [1.5–19]	6.9 [0.80–17]	8.7 [1.9–19]	9.4 [2.3–20]	<0.001
Dutch Healthy Diet Index	83 ± 15	84 ± 14	83 ± 15	84 ± 15	0.76
Dietary MGO, mg/d	4.1 ± 1.2	3.0 ± 0.61	4.0 ± 0.60	5.3 ± 1.2	<0.001
Dietary GO, mg/d	3.7 ± 1.1	2.6 ± 0.51	3.6 ± 0.53	4.8 ± 0.96	<0.001
Dietary 3-DG, mg/d	17 [12–23]	10 [8.1–13]	17 [14–20]	26 [21–33]	<0.001
Glucose status					0.01
Normal glucose metabolism, %	58	54	58	62	
Prediabetes, %	15	15	15	15	
T2DM, %	26	30	26	22	
Other types of diabetes, %	1	1	1	1	
Diabetes duration, years	4 [1–11]	4 [1–11]	5 [2–10]	4 [1–11]	0.78
Lifestyle					
Smoking, %					0.85
Never	36	36	37	35	
Former	52	51	52	53	
Current	12	13	12	12	
Waist circumference, cm	95 ± 14	96 ± 14	95 ± 14	96 ± 13	0.29
BMI	27 ± 5	27 ± 5	27 ± 5	27 ± 4	0.01
Physical activity, h/wk	13 [8.3–19]	12 [7.5–18]	13 [8.3–18]	14 [8.5–20]	<0.001
Education, %					0.22
Low	32	34	31	31	
Medium	29	30	29	28	
High	39	36	40	41	
Biological					
Fasting glucose, mmol/L	5.5 [5.1–6.4]	5.6 [5.1–6.6]	5.5 [5.1–6.4]	5.5 [5.1–6.2]	0.31
HbA1c, %	5.6 [5.4–6.2]	5.7 [5.4–6.3]	5.6 [5.4–6.2]	5.6 [5.3–6.1]	0.27
24-h systolic blood pressure, mm Hg	135 ± 18	135 ± 19	134 ± 18	135 ± 18	0.44
24-h diastolic blood pressure, mm Hg	76 ± 10	76 ± 10	76 ± 10	77 ± 10	0.33
HOMA-IR	1.4 [1.0–2.1]	1.4 [1.0–2.3]	1.4 [1.0–2.1]	1.4 [1.0–2.1]	0.35
Cholesterol, mmol/L	5.3 ± 1.2	5.2 ± 1.2	5.3 ± 1.2	5.3 ± 1.1	0.70
Total-to-HDL cholesterol ratio	3.6 ± 1.2	3.6 ± 1.1	3.6 ± 1.1	3.7 ± 1.2	0.02
Triglycerides, mmol/L	1.2 [0.90–1.7]	1.2 [0.88–1.7]	1.2 [0.88–1.7]	1.2 [0.86–1.7]	0.40
eGFR, mL/min/1.73 m^2^	88 ± 15	87 ± 15	88 ± 15	89 ± 14	0.15
History of CVD, % yes	16	16	17	16	0.87
Presence of gastrointestinal infection, %yes	12	13	11	10	0.83
Retinopathy, %	2	1	1	2	0.39
(Micro)albuminuria, %	8	8	8	8	0.92
Medication use					
Glucose-lowering medication, %yes	21	23	22	18	0.03
Antihypertensives, %yes	39	43	38	38	0.05
Lipid-modifying medication, %yes	35	38	35	33	0.11
Plasma biomarkers of low-grade inflammation					
hsCRP, µg/mL	1.2 [0.61–2.7]	1.3 [0.68–3.1]	1.2 [0.59–2.7]	1.1 [0.57–2.5]	0.01
SAA, µg/mL	3.3 [2.1–5.4]	3.4 [2.2–5.6]	3.2 [2.0–5.5]	3.2 [1.9–5.1]	0.02
sICAM1, ng/mL	338 [290–398]	341 [292–405]	339 [289–393]	336 [291–389]	0.19
IL-6, pg/mL	0.58 [0.39–0.88]	0.60 [0.41–0.89]	0.58 [0.38–0.90]	0.57 [0.38–0.86]	0.07
IL-8, pg/mL	4.1 [3.3–5.3]	4.2 [3.3–5.5]	4.0 [3.2–5.2]	4.1 [3.3–5.2]	0.01
TNF-α, pg/mL	2.2 [1.9–2.6]	2.2 [1.9–2.6]	2.2 [1.9–2.5]	2.2 [1.9–2.6]	0.27
Microvascular measurements					
Plasma biomarkers of endothelial function					
sICAM-1, ng/mL	338 [291–398]	341 [292–405]	339 [289–393]	336 [291–389]	0.23
sVCAM-1, ng/mL	427 ± 101	426 ± 103	429 ± 100	427 ± 100	0.90
sE-selectin, ng/mL	107 [75–143]	107 [72–145]	106 [74–141]	109 [78–143]	0.48
vWF, %	132 ± 48	133 ± 47	132 ± 49	132 ± 48	0.56
Retinal microvascular measurements					
CRAE, µm	142 ± 20	143 ± 20	142 ± 19	142 ± 20	0.41
CRVE, µm	214 ± 31	215 ± 32	213 ± 30	215 ± 32	0.46
Baseline arteriolar diameter, MU	115 ± 15	115 ± 15	114 ± 16	116 ± 15	0.19
Baseline venular diameter, MU	146 ± 21	147 ± 21	146 ± 21	146 ± 20	0.96
Flicker light–induced arteriolar dilation response, %	3.0 ± 2.8	3.1 ± 2.9	3.2 ± 2.8	2.9 ± 2.7	0.29
Flicker light–induced venular dilation response, %	3.9 ± 2.2	4.1 ± 2.3	3.8 ± 2.2	3.9 ± 2.2	0.06
Absolute arteriolar dilation response, delta	4.4 ± 3.6	4.4 ± 3.7	4.5 ± 3.6	4.3 ± 3.4	0.43
Absolute venular dilation response, delta	7.7 ± 4.1	7.9 ± 4.3	7.5 ± 4.0	7.5 ± 4.1	0.16
Skin microvascular measurements					
Baseline skin blood flow before heating, PU	11 ± 6.6	12 ± 7.4	11 ± 5.6	11 ± 6.7	0.07
Skin hyperemia during heating, PU	113 ± 58	114 ± 59	112 ± 55	113 ± 59	0.90
Skin hyperemic response, %	1129 ± 773	1137 ± 794	1156 ± 768	1094 ± 760	0.47
Kidney microvascular measurement					
Urinary albumin excretion, mg/24 h	6.5 [4.0–11.6]	6.3 [4.0–12]	6.5 [3.9–12]	6.9 [4.2–11]	0.85

1 Data are presented as means ± SDs for normally distributed variables, medians [IQRs] for nonnormally distributed variables, or percentage for categorical variables. *P* value of ANOVA, Kruskal-Wallis, or chi-square tests for differences between tertiles. 3-DG, 3-deoxyglucosone; CRAE, central retinal arteriolar equivalent; hsCRP, high sensitivity C-reactive protein; CRVE, central retinal venular equivalent; CVD, cardiovascular diseases; eGFR, estimated glomerular filtration rate; GO, glyoxal; sICAM-1, soluble intracellular adhesion molecule-1; MGO, methylglyoxal; MU. Measurement units; NGM, normal glucose metabolism; PU, perfusion units; SAA, serum amyloid A; sE-selectin, soluble E-selectin; sVCAM-1, soluble vascular adhesion molecule-1; T2DM, type 2 diabetes mellitus; vWF, von Willebrand factor.

2 For the tertiles, a composite score of total dicarbonyl intake (MGO, GO, and 3-DG) was created. Data were available for waist, *n* = 2791; duration of diabetes, n = 606; HOMA-IR, *n* = 2621; eGFR, *n* = 2768; HbA1c, *n* = 2785; fasting plasma glucose, *n* = 2790; history of CVD, *n* = 2777; gastrointestinal infection, *n* = 2500; hsCRP, SAA; sICAM-1, IL-8, TNF-α, sVCAM-1, e-selectin, *n* = 2766; IL-6, *n* = 2765, vWF, *n* = 2762; retinal microvascular diameters, *n* = 2438, flicker light-induced arteriolar dilation, *n* = 2104; flicker light–induced venular dilation, *n* = 2147; baseline skin blood flow and skin hyperemic response, *n* = 1357; urinary albumin excretion, *n* = 2774.

### Associations between dietary dicarbonyl intake and plasma biomarkers of low-grade inflammation

Higher intake of MGO was associated with a lower *z*-score for inflammation in the fully adjusted model [standardized β coefficient (STD β): −0.05; 95% CI: −0.09, −0.01; Table 2]. Higher intakes of GO and 3-DG were also associated with a lower *z*-score for inflammation in the crude model (STD β: −0.06; 95% CI: −0.09 to −0.02; and −0.09; −0.12 to −0.05, respectively), but these associations were attenuated after further adjustments (STD β: −0.01; 95% CI: −0.06 to 0.04; and −0.03; −0.07 to 0.002), respectively, model 3. Intake of MGO was inversely associated with each individual biomarker of inflammation except sICAM, with the strongest and statistically significant associations for hsCRP and TNF-α (STD β: −0.05; 95% CI: −0.10, −0.01; and STD β: −0.05; 95% CI: −0.10, −0.01, respectively, **Supplemental Table S3**).

**TABLE 2:** Association between dietary dicarbonyl intakes and a composite score of low-grade inflammation^1^

Model	Biomarkers of low-grade inflammation (composite score)^2^
Dietary MGO	
Crude	−0.05 (−0.09, −0.01)
1	−0.03 (−0.06, 0.01)
2	−0.05 (−0.10, −0.01)
3	−0.05 (−0.09, −0.01)
Dietary GO	
Crude	−0.06 (−0.09, −0.02)
1	−0.02 (−0.06, 0.01)
2	−0.02 (−0.07, 0.03)
3	−0.01 (−0.06, 0.04)
Dietary 3-DG	
Crude	−0.09 (−0.12, −0.05)
1	−0.05 (−0.08, −0.02)
2	−0.03 (−0.07, 0.01)
3	−0.03 (−0.07, 0.002)

1 Values are presented as STD β (95% CI) expressed as 1 SD change in composite score of low-grade inflammation per 1 SD higher dietary dicarbonyl intake. All variables were standardized for comparison. 3-DG, 3-deoxyglucosone; GO, glyoxal; MGO, methylglyoxal.

2 Composite score of low-grade inflammation consisted of the biomarkers hsCRP, SAA, sICAM-1, IL-6, IL-8 and TNF-α: model 1: adjusted for age + sex + glucose metabolism status; model 2: model 1 + BMI, total energy intake, smoking status, alcohol intake, physical activity, educational level; model 3: model 2 + triglycerides, systolic blood pressure, total cholesterol/HDL ratio, use of glucose-lowering, antihypertensive, or lipid-modifying drugs. The population consisted of 2765 individuals.

In addition, we evaluated the association between dietary dicarbonyl intake and plasma biomarkers of low-grade inflammation in an independent cohort, the CODAM study. Dicarbonyl intake in CODAM was similar to that reported for the Maastricht Study, with intakes as follows: median: 3.8; IQR: 3.1–4.6 mg/d for MGO, 3.4; 2.8–4.1 mg/d for GO, and 16; 11–22 mg/d for 3-DG, respectively. The associations between MGO, GO, and 3-DG intake and low-grade inflammation were inverse, although not statistically significant (STD β: −0.05; 95% CI: −0.16, 0.05 for MGO; −0.09; −0.22, 0.04 for GO; and −0.01; −0.11, 0.09 for 3-DG, respectively, model 3, **Supplemental Tables S4** and **S5**).

### Associations between dietary dicarbonyl intake and plasma biomarkers of endothelial function

Intakes of MGO and GO were not associated with the *z*-score for endothelial function (STD β: −0.01; 95% CI:−0.06, 0.03 and 0.03; −0.03, 0.07 for MGO and GO, respectively, model 3, Table 3). Higher intake of 3-DG was associated with a lower *z*-score for endothelial function in the crude model: STD β: −0.05; 95% CI: −0.09, −0.01. However, this association was attenuated after further adjustment (STD β: −0.01; 95% CI: −0.05, 0.02; model 3). The associations of dietary MGO, GO, and 3-DG with individual biomarkers of endothelial function were also not statistically significant (**Supplemental Table S6**).

**TABLE 3:** Associations between dietary dicarbonyls and measures of microvascular function^1^

	Biomarkers of endothelial function^2^	CRAE	CRVE	Retinal arteriolar average dilation^3^	Retinal venular average dilation^3^	Heat-induced skin hyperemia^4^	Urinary albumin excretion^5^
Dietary MGO							
Crude	0.003 (−0.03, 0.04)	−0.01 (−0.04, 0.05)	0.02 (−0.02, 0.06)	−0.004 (−0.05, 0.04)	−0.05 (−0.09, −0.01)	−0.05 (−0.11, −0.01)	0.05 (0.02, 0.09)
1	0.002 (−0.03, 0.04)	0.03 (−0.01, 0.07)	0.04 (−0.001, 0.08)	−0.02 (−0.06, 0.03)	−0.05 (−0.09, −0.004)	−0.02 (−0.07, 0.03)	0.05 (0.01, 0.08)
2	−0.02 (−0.06, 0.03)	0.04 (−0.01, 0.09)	0.04 (−0.01, 0.09)	−0.01 (−0.07, 0.04)	−0.07 (−0.12, −0.02)	−0.02 (−0.08, 0.05)	0.04 (−0.001, 0.09)
3	−0.01 (−0.06, 0.03)	0.04 (−0.01, 0.09)	0.04 (−0.01, 0.09)	−0.01 (−0.06, 0.05)	−0.07 (−0.12, −0.01)	−0.02 (−0.08, 0.05)	0.04 (−0.001, 0.09)
Dietary GO							
Crude	0.007 (−0.03, 0.05)	−0.02 (−0.06, 0.02)	−0.01 (−0.05, 0.03)	−0.02 (−0.06, 0.03)	−0.03 (−0.07, 0.02)	−0.02 (−0.07, 0.04)	−0.01 (−0.05, 0.03)
1	0.02 (−0.02, 0.06)	−0.001 (−0.04, 0.04)	0.000 (−0.04, 0.04)	−0.04 (−0.08, 0.01)	−0.03 (−0.07, 0.02)	0.01 (−0.05, 0.06)	−0.01 (−0.04, 0.03)
2	0.012 (−0.03, 0.08)	−0.01 (−0.07, 0.05)	0.000 (−0.06, 0.06)	−0.08 (−0.14, −0.01)	−0.07 (−0.14, −0.01)	0.03 (−0.05, 0.10)	−0.04 (−0.10, 0.01)
3	0.03 (−0.03, 0.07)	−0.01 (−0.07, 0.05)	0.002 (−0.06, 0.06)	−0.07 (−0.14, −0.01)	−0.07 (−0.13, 0.001)	0.03 (−0.05, 0.10)	−0.05 (−0.10, 0.01)
Dietary 3-DG							
Crude	−0.05 (−0.09, −0.01)	−0.03 (−0.07, 0.01)	−0.01 (−0.05, 0.03)	−0.01 (−0.05, 0.03)	0.003 (−0.04, 0.05)	0.02 (−0.03, 0.08)	−0.02 (−0.06, 0.02)
1	−0.03 (−0.06, 0.01)	−0.02 (−0.05, 0.03)	0.003 (−0.04, 0.04)	−0.03 (−0.07, 0.01)	0.002 (−0.04, 0.05)	0.03 (−0.03, 0.08)	−0.01 (−0.04, 0.03)
2	−0.01 (−0.05, 0.03)	−0.01 (−0.05, 0.03)	0.01 (−0.03, 0.05)	−0.03 (−0.08, 0.02)	−0.003 (−0.05, 0.05)	0.01 (−0.05, 0.07)	−0.004 (−0.04, 0.04)
3	−0.01 (−0.05, 0.02)	−0.01 (−0.05, 0.04)	0.01 (−0.03, 0.06)	−0.03 (−0.08, 0.02)	−0.004 (−0.05, 0.04)	0.01 (−0.05, 0.07)	−0.01 (−0.05, 0.03)

1 Values are presented as STD β (95% CI) expressed as 1-SD change in the outcome variables per 1-SD higher dietary dicarbonyl. All variables were standardized for comparison. Model 1, adjusted for age + sex + glucose metabolism status; model 2: model 1 + BMI, total energy intake, smoking status, alcohol intake, physical activity, educational level; model 3: model 2 + triglycerides, systolic blood pressure, total cholesterol/HDL ratio, use of glucose-lowering, antihypertensive, or lipid-modifying drugs. The endothelial function population consisted of 2745 individuals, the CRAE/CRVE population consisted of 2438 individuals, the retinal dilation population consisted of 2078 individuals, the heat-induced skin hyperemia population consisted of 1357 individuals, the urinary albumin excretion population consisted of 2774 individuals. 3-DG, 3-deoxyglucosone; CRAE, central retinal arteriolar equivalent; CRVE, central retinal venular equivalent; GO, glyoxal; MGO, methylglyoxal.

2 Composite score of plasma biomarkers of endothelial function, consisted of the biomarkers sVCAM-1, sICAM-1, sE-selectin and vWF.

3 Baseline retinal venular diameter before flicker light was associated with dietary MGO intake (see Supplemental Table S1), thus absolute change of retinal venular dilation over baseline (delta) was used as outcome in the association between MGO intake and retinal venular dilation, and percentage change over baseline was used as outcome for the other associations.

4 Baseline skin hyperemia was significantly associated with dietary 3-DG (see Supplemental Table S1), so for the association between dietary 3-DG and skin hyperemia the average total heating response in the arm (in PU, in the interval 2–25 min) was used as outcome measure with additional adjustment for baseline skin hyperemia in all models, instead of the percentage change.

5 Urinary albumin excretion was ln-transformed. Higher urinary albumin excretion indicates worse microvascular function.

### Association between dietary dicarbonyl intake and retinal microvascular function

Higher intake of MGO was associated with impaired flicker light–induced retinal venular dilation in all models (STD β absolute change over baseline: −0.07; 95% CI: −0.12, −0.01, model 3, Table 3), but not with retinal arteriolar dilation (STD β percentage change over baseline: −0.01; 95% CI: −0.06, 0.05; model 3). Results were similar when the percentage change of retinal venular dilation was used as an outcome (data not shown). Higher intake of GO was associated with impaired flicker light–induced retinal arteriolar dilation after full adjustment (STD β percentage change over baseline: −0.07; 95% CI: -0.14, −0.01; model 3, Table 3), but not with retinal venular dilation (STD β percentage change over baseline: −0.07; 95% CI:−0.13, 0.001; model 3). Intake of 3-DG was not associated with flicker light–induced arteriolar and venular dilation (STD β: −0.03 (−0.08, 0.02) and −0.004 (−0.05, 0.04), respectively, model 3). Intakes of MGO, GO, and 3-DG were not associated with retinal arteriolar diameters (CRAE) or retinal venular diameters (CRVE) (STD β: 0.04; 95% CI: −0.01, 0.09 and 0.04; −0.01, 0.09 for MGO; −0.01; −0.07, 0.05 and 0.002; −0.06, 0.06 for GO; and −0.01; −0.05, 0.04 and 0.01; −0.03, 0.06; for 3-DG, respectively, model 3).

### Associations between dietary dicarbonyl intake and heat-induced skin hyperemia

Higher intake of MGO was associated with impaired heat-induced skin hyperemia in the crude model (STD β percentage change over baseline: −0.05; 95% CI: −0.11, −0.01; Table 3), but this result was attenuated after further adjustment (STD β: −0.02; −0.08, 0.05, model 3). Intake of GO and 3-DG were not associated with heat-induced skin hyperemia [STD β percentage change over baseline: 0.03; 95% CI: −0.05, 0.10 for GO; and STD β absolute average heating response: 0.01; 95% CI: −0.05, 0.07 for 3-DG (model 3)].

### Associations between dietary dicarbonyl intake and urinary albumin excretion

Higher intake of MGO was associated with higher excretion of urinary albumin in the crude and in the age-, sex-, and glucose metabolism status adjusted models (STD β: 0.05; 95% CI: 0.01, 0.08; model 1, Table   3), but did not remain statistically significant after further adjustment (STD β: 0.04; 95% CI: −0.001, 0.09; model 3). Intake of GO and 3-DG were not associated with urinary albumin excretion (STD β: −0.05; 95% CI: −0.10, 0.01; for GO and −0.01; −0.05, 0.03; for 3-DG, respectively; model 3).

### Interaction analyses

Glucose metabolism status modified the associations of dietary GO with retinal venular diameter, retinal arteriolar dilation, and urinary albumin excretion (*P*-interaction 0.02–0.07). Stratified analyses revealed a significant association between higher GO intake and impaired retinal arteriolar dilation in individuals with type 2 diabetes (normal glucose metabolism STD β: −0.06; 95% CI: −0.14, 0.03; prediabetes: 0.02; −0.16, 0.20; and type 2 diabetes: −0.13; −0.26, −0.01, **Supplemental Table S7**). Similarly, higher GO intake was associated with lower urinary albumin excretion in individuals with type 2 diabetes only (normal glucose metabolism STD β: 0.04; 95% CI: −0.03, 0.10; prediabetes: −0.07; −0.21, 0.08; and type 2 diabetes: −0.13; −0.26, −0.01, respectively.

Additionally, glucose metabolism status modified the associations of dietary 3-DG with retinal venular diameter, with retinal venular dilation, skin hyperemia, and urinary albumin excretion (*P*-interaction 0.01–0.09). Stratified analyses revealed no significant associations in any of the glucose metabolism groups (Supplemental Table S7).

Sex modified the association between dietary GO and retinal arteriolar dilation (*P*-interaction = 0.06), but stratified analyses revealed no significant association for males or females (data not shown). eGFR did not modify any of the associations.

### Additional analyses

We explored the contribution of several main food groups (≥5% of daily dicarbonyl intake) to the strength of the observed associations. The strength of the above-reported association between dietary MGO intake and low-grade inflammation remained similar after adjustment for MGO intake from any individual food group but was no longer statistically significant after additional adjustment for MGO intake from either coffee or cookies and bakery products (Table 4). The above-reported association between dietary MGO intake and retinal venular dilation (STD β: −0.07; 95% CI:−0.12, −0.01) was attenuated and lost statistical significance after adjustment for MGO intake from coffee (STD β: −0.04; 95% CI:−0.12, 0.05; Table 4).

**TABLE 4:** Association of dietary MGO with low-grade inflammation and retinal venular dilation additionally adjusted for main food groups^1^

Dietary MGO model	% of MGO intake^3^	Biomarkers of low-grade inflammation (composite score)^2^	Retinal venular dilation (delta)
			
Fully adjusted model (model 3)^4^		−0.05 (−0.09, −0.01)	−0.07 (−0.12, −0.01)
Model 3 + coffee	26	−0.05 (−0.12, 0.01)	−0.04 (−0.12, 0.05)
Model 3 + bread	23	−0.05 (−0.09, −0.01)	−0.07 (−0.12, −0.01)
Model 3 + vegetables and legumes	10	−0.05 (−0.09, −0.004)	−0.07 (−0.13, −0.02)
Model 3 + meat	9	−0.05 (−0.09, −0.01)	−0.07 (−0.12, −0.01)
Model 3 + cookies and bakery products	8	−0.04 (−0.09, 0.003)	−0.07 (−0.13, −0.01)
Model 3 + ready-made	5	−0.05 (−0.09, −0.01)	−0.07 (−0.12, −0.01)

1 Values are presented as STD β (95% CI) expressed as 1-SD change in composite score of low-grade inflammation or 1-SD change in flicker light–induced retinal venular dilation (delta) per 1 SD higher dietary MGO intake. All variables were standardized for comparison. MGO, methylglyoxal.

2 Composite score of low-grade inflammation consisted of the biomarkers hsCRP, SAA, sICAM-1, IL-6, IL-8 and TNF-α.

3 Percentage of MGO intake via each food group [as reported in (4)].

4 Fully adjusted model (model 3): adjusted for age, sex, glucose metabolism status, BMI, total energy intake, smoking status, alcohol intake, physical activity, educational level, triglycerides, systolic blood pressure, total cholesterol/HDL ratio, use of glucose-lowering-, antihypertensive- or lipid-modifying drugs. Additionally, sequentially adjusted for MGO intake via each of the separate food groups that contributed ≥5% to total MGO intake.

In our sensitivity analyses, the observed association between dietary MGO and biomarkers of low-grade inflammation remained of similar strength after the majority of sensitivity analyses. However, statistical significance was lost when we adjusted for physical activity using ActivPAL data instead of the CHAMPS questionnaire, when we excluded individuals with gastrointestinal tract infection, or when we excluded individuals with previously diagnosed type 2 diabetes, results that were most likely due to limited power (**Supplemental Table S8**). The observed association between dietary MGO and retinal venular dilation remained of similar strength but was no longer statistically significant after exclusion of individuals with previously diagnosed type 2 diabetes or adjustment for 24-h ambulatory blood pressure instead of office blood pressure, again probably because of limited power (**Supplemental Table S9**).

## Discussion

In this population-based cohort, we observed that higher habitual intake of MGO was associated with lower low-grade inflammation scores, but not consistently with features of microvascular function. GO or 3-DG intakes were not consistently associated with low-grade inflammation or microvascular function.

The association between higher MGO intake and lower low-grade inflammation scores was somewhat unexpected, considering that authors of animal studies have reported that high amounts of pure oral MGO increases vascular inflammation (8, 10). Nevertheless, our results were corroborated by the observation of a similar inverse, although not significant, association between MGO intake and inflammation in the independent CODAM cohort. This association between higher MGO intake and a lower degree of inflammation may be attributable to potential antioxidative effects of MGO, which were reported both in vitro (14, 16, 39) and in an animal study (11). These antioxidative effects are thought to occur via an MGO-induced defense mechanism involving the KEAP1-Nrf2 pathway (14, 16, 39). The transcription factor Nrf2 is a key regulator in oxidative stress, increasing the production of specific antioxidant enzymes and glyoxalase-1, both involved in cellular protection against glycation. Thus, small increases in plasma MGO after exogenous exposure to food-derived MGO may induce the defense system against glycation and thereby prevent protein damage caused by glycation. Another mechanism behind these antioxidative effects could be via formation of the methylglyoxal-derived hydroimidazolone MG-H3 (40). MG-H3 is one of the adducts that is formed after modification of the amino acid arginine by MGO and was found to possess antioxidant properties in vitro (41).

In the current study, MGO intake was not significantly associated with most microvascular features in the retina, skin, plasma, and kidney. We found that higher MGO intake was associated only with impaired retinal venular (but not arteriolar) dilation, suggesting a detrimental effect of MGO intake on retinal venular function. Venular dilation after flicker-light stimulation is an endothelium-dependent response, and this observation hence points towards dysfunction of retinal venular endothelium (21, 25). MGO might contribute to impaired vasodilation via dysregulation of endothelial NO synthase (eNOS), leading to reduced nitric oxide (NO) production and thus reduced vasoreactivity (1, 42–44). In accordance with this explanation, studies in animals have shown that exogenous administration of MGO induces vascular changes, including impaired vasodilation (8, 45), reduced NO bioavailability, and endothelial dysfunction (8). Moreover, oral MGO administration induced retinopathy-like changes, such as pericyte loss, formation of acellular capillaries, microglial activation, and early neuronal dysfunction (46). A possible explanation of why we only observed an impairment of retinal venular dilation and not of other microvascular features is that retinal vessel dilation is a sensitive marker of generalized microvascular function (47, 48), and the association with microvascular function might only be detected for this outcome. However, we cannot exclude the possibility that the association between higher MGO intake and impaired retinal venular dilation is a chance finding due to multiple testing. Therefore, the association with retinal venular dilation has to be interpreted with caution, and this observation requires confirmation in other studies.

Some associations were modified by glucose metabolism status, but after stratification for glucose metabolism group, there was no clear pattern, so this may be a chance finding.

Strengths of this study include the availability of a large human cohort deeply phenotyped with validated, state-of-the-art techniques to assess inflammation and microvascular function in various organs, and extensive assessment of potential confounders. Another strength is the application of the most extensive food composition database for dicarbonyls currently available worldwide, in which the highly sensitive UPLC-MS/MS was used for quantification of MGO, GO, and 3-DG, and which was specifically developed to match the comprehensive 253-item FFQ used in this cohort. Moreover, we were able to verify the association between dietary dicarbonyl intake and low-grade inflammation in an independent cohort. This study also has certain limitations. Food items that are high in MGO may also contain other anti-inflammatory or antioxidant components, for example, caffeine in coffee (49). Because coffee was the main contributor to MGO intake (4), we cannot exclude the possibility that the presence of caffeine or other anti-inflammatory components from coffee overruled the potential adverse effects of MGO. Nevertheless, additional adjustment for MGO intake from coffee did not change the strength of the association between MGO intake and inflammation. In addition, the food composition database for dicarbonyls contains average values of dicarbonyls in foods and is, therefore, unable to take effects of individual industrial (e.g., different production methods for different brands) and household food preparation (e.g., personal preferences regarding frying conditions) on dicarbonyl intake into account. Nevertheless, the FFQ includes questions on preferred methods of food preparation, such as boiling and frying, and our dicarbonyl database contains information on foods prepared using various common preparation methods (19). Although we cannot exclude that measurement error has occurred in the estimation of dietary dicarbonyl intake, these errors are expected to be random and random measurement error in dietary dicarbonyl intakes (our exposure variables) would lead to regression to the null and thus underestimation of true associations (50). Another limitation is the cross-sectional design of the study, which does not allow the assessment of causality. In addition, although we carefully adjusted for a large set of potential confounders, residual confounding remains a possibility.

In conclusion, higher habitual intake of MGO was associated with a lower degree of low-grade inflammation but was not consistently associated with microvascular function. This study is to our knowledge the first to report on associations between dietary MGO and health outcomes. This observed, presumably beneficial, association warrants further investigation.

## Supplementary Material

nqac195_Supplemental_File

## Data Availability

The data that underlie the findings of this study are available from the corresponding author and the Maastricht Study Management Team (research.dms@mumc.nl) upon reasonable request.

## Notes

This study was partly funded by the Netherlands Food and Consumer Product Safety Authority (NVWA). This project has received cofunding from the PPP Allowance made available by Health∼Holland, Top Sector Life Sciences & Health, to stimulate public–private partnerships, under the umbrella of the European Joint Programming Initiative “A Healthy Diet for a Healthy Life” (JPI HDHL) and of the ERA-NET Cofund HDHL INTIMIC (GA N 727565 of the EU Horizon 2020 Research and Innovation Programme.) The Maastricht Study was supported by the European Regional Development Fund via OP‐Zuid, the Province of Limburg, the Dutch Ministry of Economic Affairs (grant 31O.041), Stichting De Weijerhorst (Maastricht, the Netherlands), the Pearl String Initiative Diabetes (Amsterdam, the Netherlands), the Cardiovascular Center (CVC; Maastricht), CARIM School for Cardiovascular Diseases (Maastricht), CAPHRI Care and Public Health Research Institute (Maastricht), NUTRIM School for Nutrition and Translational Research in Metabolism (Maastricht), Stichting Annadal (Maastricht), and the Health Foundation Limburg (Maastricht), Perimed (Järfalla, Sweden), Imedos Systems GmbH (Jena, Germany), and by unrestricted grants from Janssen‐Cilag B.V. (Tilburg, the Netherlands), Novo Nordisk Farma B.V. (Alphen aan den Rijn, the Netherlands), and Sanofi‐Aventis Netherlands B.V. (Gouda, the Netherlands). These sources of support did not have any role in the study or this publication.

Supplemental Tables S1–9 and Supplemental Figure S1 are available from the Supplementary data link in the online posting of the article and from the same link in the online table of contents at https://academic.oup.com/ajcn/.

Abbreviations used: 3-DG, 3-deoxyglucosone; AGE, advanced glycation endproduct; CODAM, Cohort on Diabetes and Atherosclerosis Maastricht; CRAE, central retinal arteriolar equivalent; CRVE, central retinal venular equivalent; DHD15, Dutch Healthy Diet Index 2015; DVA, dynamic vessel analyzer; eGFR, estimated glomerular filtration rate; eNOS, endothelial NO synthase; EPIC, European Prospective Investigation into Cancer and Nutrition; GMS, glucose metabolism status; GO, glyoxal; HbA1c, glycosylated hemoglobin; hsCRP, high-sensitivity C-reactive protein; MG-Hs, methylglyoxal-derived hydroimidazolones; MGO, methylglyoxal; MU, measurement units; NEVO, Dutch Food Composition Table; NO, nitric oxide; PU, per unit; SAA, serum amyloid A; sE-selectin, soluble E-selectin; sICAM-1, soluble intracellular adhesion molecule-1; STD β, standardized β coefficient; sVCAM-1, soluble vascular adhesion molecule-1; UPLC, ultraperformance liquid chromatography; vWF, von Willebrand factor.
