# Supplementary material for: Habitual intake of dietary methylglyoxal is associated with less low-grade inflammation: the Maastricht Study
Source: Am J Clin Nutr. 2022 Sep 7;116(6):1715–28. doi: 10.1093/ajcn/nqac195 (PMC9761753; doi:10.1093/ajcn/nqac195)
Supplement: nqac195_Supplemental_File [file nqac195_supplemental_file.docx]

**Supplementary tables**

**Supplementary Table 1** - Associations of dietary dicarbonyl intakes with baseline retinal vessel diameters and with baseline skin blood flow ^1^

|  | **Retinal arteriolar baseline diameter (MU)** | **Retinal venular baseline diameter (MU)** | **Skin baseline blood flow (PU)** |
| --- | --- | --- | --- |
| **Dietary MGO** | Std. β (95% CI) | Std. β (95% CI) | Std. β (95% CI) |
| Crude | 0.02 (-0.02, 0.06) | 0.05 (0.01, 0.09) | 0.001 (-0.05, 0.05) |
| 1 | 0.03 (-0.02, 0.07) | 0.05 (0.001, 0.09) | -0.001 (-0.05, 0.05) |
| 2 | 0.02 (-0.04, 0.07) | 0.06 (0.01, 0.12) | -0.02 (-0.08, 0.05) |
| 3 | 0.01 (-0.04, 0.07) | 0.06 (0.01, 0.12) | -0.02 (-0.08, 0.05) |
|  |  |  |  |
| **Dietary GO** | Std. β (95% CI) | Std. β (95% CI) | Std. β (95% CI) |
| Crude | 0.02 (-0.02, 0.06) | -0.006 (-0.05, 0.04) | -0.01 (-0.06, 0.05) |
| 1 | 0.03 (-0.02, 0.07) | -0.01 (-0.05, 0.04) | -0.01 (-0.06, 0.04) |
| 2 | 0.03 (-0.04, 0.10) | 0.01 (-0.06, 0.07) | -0.05 (-0.14, 0.03) |
| 3 | 0.02 (-0.04, 0.09) | 0.01 (-0.06, 0.08) | -0.06 (-0.14, 0.03) |
|  |  |  |  |
| **Dietary 3-DG** | Std. β (95% CI) | Std. β (95% CI) | Std. β (95% CI) |
| Crude | 0.002 (-0.04, 0.05) | -0.03 (-0.07, 0.02) | -0.04 (-0.09, 0.01) |
| 1 | 0.01 (-0.04, 0.05) | -0.03 (-0.07, 0.02) | -0.05 (-0.10, 0.01) |
| 2 | 0.01 (-0.04, 0.06) | -0.01 (-0.06, 0.04) | -0.07 (-0.13, -0.01) |
| 3 | 0.02 (-0.03, 0.07) | -0.01 (-0.06, 0.04) | -0.07 (-0.12, -0.01) |

^1^ Standardized betas (β) were expressed as 1 SD change in outcome variable per 1 SD higher dietary dicarbonyl intake. These analyses were not of primary interest, but were needed to determine what outcome variable to use for retina and skin analyses.
Model 1: adjusted for age + sex + glucose metabolism status.
Model 2: model 1 + BMI, total energy intake, smoking status, alcohol intake, physical activity, educational level.
Model 3: model 2 + triglycerides, systolic blood pressure, total cholesterol/HDL ratio, use of glucose-lowering-, antihypertensive- or lipid-modifying drugs.
3-DG, 3-deoxyglucosone, GO, glyoxal, MGO, methylglyoxal, MU, measurement units, PU, perfusion units.

**Supplementary Table 2** - Comparison of population characteristics of the various subpopulations ^1^

| Characteristics | Total included population in main analyses (n=2792) | Subpopulations | | | | | | | |
| --- | --- | --- | --- | --- | --- | --- | --- | --- | --- |
|  |  | Excluded population (n=659) | Low-grade inflammation population (n=2765) | Endothelial function population (n=2745) | Retinal vessel diameter population (n=2438) | Retinal vessel dilation population (n=2078) | Skin hyperemia population (n=1357) | Urinary albumin excretion population (n=2774) | P_excluded_ |
| **Demographics** | | | | | | | | | |
| Age (years) | 60 ± 8 | 60 ± 9 | 60 ± 8 | 60 ± 8 | 60 ± 8 | 60 ± 8 | 60 ± 8 | 60 ± 8 | 0.63 |
| Sex (% male) | 50 | 57 | 50 | 50 | 50 | 51 | 51 | 50 | 0.001 |
| **Glucose metabolism status** | | | | | |  |  |  | <0.001 |
| Normal glucose metabolism (%) | 58 | 47 | 58 | 58 | 58 | 59 | 56 | 58 |  |
| Prediabetes (%) | 15 | 14 | 15 | 15 | 15 | 15 | 16 | 15 |  |
| Type 2 diabetes mellitus (%) | 26 | 37 | 26 | 26 | 27 | 25 | 27 | 26 |  |
| Other types of diabetes (%) | 1 | 2 | 1 | 1 | 1 | 1 | 1 | 1 |  |
| Diabetes duration (years) | 4 [1-11] | 6 [2-11] | 4 [1-11] | 4 [1-11] | 4 [1-11] | 4 [1-10] | 4 [1-11] | 4 [1-11] | 0.05 |
| **Lifestyle** | | | | | | | | | |
| Smoking (%) |  |  |  |  |  |  |  |  | <0.001 |
| - Never | 36 | 28 | 36 | 36 | 36 | 36 | 34 | 36 |  |
| - Former | 52 | 50 | 52 | 52 | 52 | 52 | 55 | 52 |  |
| - Current | 12 | 22 | 12 | 12 | 12 | 11 | 11 | 12 |  |
| Waist circumference (cm) | 95 ± 14 | 98 ± 14 | 95 ± 14 | 95 ± 14 | 95 ± 14 | 95 ± 14 | 96 ± 13 | 95 ± 14 | <0.001 |
| BMI (kg/m^2^) | 27 ± 5 | 28 ± 5 | 27 ± 5 | 27 ± 5 | 27 ± 5 | 27 ± 4 | 27 ± 4 | 27 ± 5 | 0.01 |
| Physical activity (h/week) | 13 [8.3-19] | 13 [7.8-18] | 13 [8.3-19] | 13 [8.3-19] | 13 [8-18] | 13 [8-19] | 13 [8.3-18] | 13 [8.3-19] | 0.55 |
| Education (%) |  |  |  |  |  |  |  |  | <0.001 |
| - Low | 32 | 41 | 32 | 32 | 32 | 31 | 32 | 32 |  |
| - Medium | 29 | 27 | 29 | 29 | 29 | 29 | 28 | 29 |  |
| - High | 39 | 23 | 40 | 40 | 39 | 40 | 40 | 39 |  |
| **Biological** | | | | | |  |  |  |  |
| Fasting glucose (mmol/L) | 5.5 [5.1-6.4] | 5.8 [5.2-7.2] | 5.5 [5.1-6.4] | 5.5 [5.1-6.4] | 5.5 [5.1-6.4] | 5.5 [5.0-6.4] | 5.5 [5.0-6.5] | 5.5 [5.1-6.4] | <0.001 |
| HbA1c, % | 5.6 [5.4-6.2] | 5.8 [5.4-6.5] | 5.6 [5.3-6.1] | 5.6 [5.4-6.1] | 5.6 [5.4-6.2] | 5.6 [5.4-6.1] | 5.7 [5.4-6.3] | 5.6 [5.4-6.2] | <0.001 |
| 24-h Systolic blood pressure (mmHg) | 135 ± 18 | 136 ± 19 | 135 ± 18 | 135 ± 18 | 135 ± 18 | 135 ± 18 | 136 ± 18 | 135 ± 18 | 0.15 |
| 24-h Diastolic blood pressure (mmHg) | 76 ± 10 | 76 ± 10 | 76 ± 10 | 76 ± 10 | 76 ± 10 | 76 ± 10 | 76 ± 9.6 | 76 ± 10 | 0.90 |
| HOMA-IR | 1.4 [1.0-2.1] | 1.5 [1.0-2.2] | 1.4 [1.0-2.1] | 1.4 [1.0-2.1] | 1.4 [0.97-2.11] | 1.4 [1.0-2.1] | 1.4 [0.94-2.1] | 1.4 [1.0-2.1] | 0.01 |
| Cholesterol (mmol/L) | 5.3 ± 1.2 | 5.1 ± 1.2 | 5.2 ± 1.2 | 5.2 ± 1.2 | 5.2 ±1.2 | 5.3 ± 1.2 | 5.3 ± 1.2 | 5.3 ± 1.2 | 0.83 |
| Total-to-HDL cholesterol ratio | 3.6 ± 1.2 | 3.8 ± 1.2 | 3.6 ± 1.2 | 3.6 ± 1.2 | 3.6 ± 1.1 | 3.6 ± 1.2 | 3.6 ± 1.1 | 3.6 ± 1.2 | 0.02 |
| Triglycerides (mmol/L) | 1.2 [0.87-1.7] | 1.2 [0.90-1.8] | 1.2 [0.88-1.7] | 1.2 [0.89-1.7] | 1.2 [0.87-1.7] | 1.2 [0.87-1.7] | 1.2 [0.88-1.7] | 1.2 [0.88-1.7] | 0.23 |
| eGFR (mL/min/1.73 m^2^) | 88 ± 15 | 88 ± 16 | 88 ± 15 | 88 ± 15 | 88 ± 15 | 88 ± 14 | 88±15 | 88 ± 15 | 0.92 |
| Medical history of CVD (% yes) | 16 | 20 | 16 | 16 | 16 | 15 | 17 | 16 | 0.03 |
| Medical history of gastrointestinal disease (%yes) | 12 | 14 | 11 | 11 | 12 | 12 | 12 | 12 | 0.49 |
| Retinopathy (%) | 2 | 3 | 1 | 2 | 2 | 2 | 2 | 2 | 0.77 |
| (Micro)albuminuria (%) | 8 | 12 | 8 | 8 | 8 | 8 | 8 | 8 | 0.001 |
| **Medication use** | | | | | | | | | |
| Glucose-lowering medication (%yes) | 21 | 33 | 21 | 21 | 22 | 20 | 22 | 21 | <0.001 |
| Anti-hypertensives (%yes) | 39 | 45 | 39 | 39 | 40 | 37 | 41 | 39 | 0.01 |
| Lipid-modifying medication (%yes) | 35 | 41 | 36 | 36 | 36 | 34 | 38 | 36 | 0.01 |
| **Dietary intake** | | | | | |  |  |  |  |
| Energy intake (kcal/day) | 2184 ± 602 | 2165 ± 637 | 2184 ± 599 | 2183 ± 599 | 2180 ± 605 | 2177 ± 596 | 2179 ± 585 | 2184 ± 601 | 0.56 |
| Carbohydrate, total (g/day) | 233 ± 70 | 231 ± 76 | 233 ± 70 | 233 ± 70 | 234 ± 70 | 233 ± 69 | 232 ± 68 | 233 ± 70 | 0.52 |
| Fat, total (g/day) | 84 ± 31 | 85 ± 31 | 84 ± 31 | 84 ± 31 | 84 ± 31 | 84 ± 31 | 84 ± 30 | 84 ± 31 | 0.84 |
| Protein (g/day) | 86 ± 23 | 84 ± 24 | 86 ± 23 | 86 ± 23 | 86±23 | 86 ± 23 | 86 ± 22 | 86 ± 23 | 0.14 |
| Fiber (g/day) | 27 ± 8.1 | 26 ± 8.5 | 27 ± 8.1 | 27 ± 8.1 | 27±8.1 | 27 ± 8.0 | 27 ± 7.8 | 27 ± 8.1 | 0.10 |
| Alcohol intake (g/day) | 8.5 [1.5-19] | 6.7 [0.79-19] | 8.6 [1.6-19] | 8.6 [1.5-19] | 8.6 [1.6-19] | 8.7 [1.6-19] | 8.8 [1.5-20] | 8.6 [1.6-19] | 0.07 |
| Dietary MGO (mg/day) | 4.1 ± 1.2 | 4.0 ± 1.3 | 4.1 ± 1.2 | 4.1 ± 1.2 | 4.1±1.2 | 4.1 ± 1.2 | 4.1 ± 1.2 | 4.1 ± 1.2 | 0.40 |
| Dietary GO (mg/day) | 3.7 ± 1.1 | 3.6 ± 1.2 | 3.7 ± 1.1 | 3.7 ± 1.1 | 3.7±1.1 | 3.7 ± 1.1 | 3.7 ± 1.1 | 3.7 ± 1.1 | 0.19 |
| Dietary 3-DG (mg/day) | 17 [12-23] | 16 [11-23] | 17 [12-23] | 17 [12-23] | 17 [11-23] | 17 [12-23] | 17 [12-23] | 17 [12-23] | 0.16 |
| Dutch Healthy Diet Index | 83 ± 15 | 82 ±14 | 83 ± 15 | 83 ± 15 | 84±15 | 84 ± 15 | 83±15 | 83 ± 15 | 0.04 |
| **Plasma biomarkers of inflammation** | | | | | | | | | |
| hsCRP (µg/ml) | 1.2 [0.61-2.7] | 1.4 [0.65-3.1] | 1.2 [0.61-2.7] | 1.2 [0.61-2.7] | 1.2 [0.60-2.6] | 1.2 [0.61-2.7] | 1.2 [0.63-2.7] | 1.2 [0.61-2.7] | 0.02 |
| SAA (µg/ml) | 3.3 [2.1-5.4] | 3.3 [2.0-5.6] | 3.3 [2.1-5.4] | 3.3 [2.1-5.4] | 3.3 [2.1-5.4] | 3.3 [2.1- 5.4] | 3.3 [2.2-5.5] | 3.3 [2.1-5.4] | 0.85 |
| sICAM1 (ng/ml) | 338 [291-398] | 345 [296-415] | 338 [290-398] | 338 [291-398] | 339 [293-399] | 338 [291- 398] | 344 [297-401] | 338 [290-398] | 0.01 |
| IL-6 (pg/ml) | 0.58 [0.39-0.88] | 0.66 [0.43-1.0] | 0.58 [0.39-0.88] | 0.58 [0.39-0.88] | 0.58 [0.39-0.88] | 0.57 [0.38-0.87] | 0.58 [0.39-0.86] | 0.58 [0.39-0.88] | <0.001 |
| IL-8 (pg/ml) | 4.1 [3.3-5.3] | 4.3 [3.5-5.6] | 4.1 [3.3-5.3] | 4.1 [3.3-5.2] | 4.1 [3.2-5.3] | 4.0 [3.2-5.2] | 4.1 [3.3-5.2] | 4.1 [3.3-5.3] | <0.001 |
| TNF-α (pg/ml) | 2.2 [1.9-2.6] | 2.2 [1.9-2.6] | 2.2 [1.9-2.6] | 2.2 [1.9-2.6] | 2.2 [1.9-2.6] | 2.2 [1.9-2.6] | 2.2 [1.9-2.5] | 2.2 [1.9-2.6] | 0.02 |
| **Microvascular measurements** | | | | | | | | | |
| Plasma biomarkers of endothelial function | | | | | | | | | |
| sICAM-1 (ng/ml) | 338 [291-398] | 345 [296-415] | 338 [290-398] | 338 [290-398] | 339 [293-399] | 338 [291- 398] | 344 [297-401] | 338 [290-398] | 0.01 |
| sVCAM-1 (ng/ml) | 427 ± 101 | 433 ±105 | 428 ± 101 | 429 ± 101 | 428 ± 100 | 428 ± 103 | 435 ± 101 | 427 ± 101 | 0.21 |
| sE-selectin (ng/ml) | 107 [75-143] | 112 [78-151] | 107 [75-143] | 107 [75-143] | 108 [75-144] | 107 [75-142] | 107 [75-143] | 107 [75-143] | 0.01 |
| vWF (%) | 132 ± 48 | 136 ± 51 | 132 ± 48 | 132 ± 48 | 132 ± 48 | 124 [99-157] | 132 ± 47 | 132 ± 48 | 0.07 |
| Retinal microvascular measurements | | | | | | | | | |
| CRAE (µm) | 142 ± 20 | 142 ± 23 | 142 ± 19 | 142 ± 20 | 142 ± 20 | 143 ± 19 | 143 ± 20 | 142 ± 20 | 0.62 |
| CRVE (µm) | 214 ± 31 | 215 ± 33 | 214 ± 31 | 214 ± 31 | 214 ± 31 | 215 ± 31 | 215 ± 32 | 214 ± 31 | 0.94 |
| Baseline arteriolar diameter (MU) | 115 ± 15 | 117 ±16 | 115 ± 16 | 115 ± 15 | 115 ± 16 | 115 ± 15 | 116 ± 16 | 115 ± 15 | 0.02 |
| Baseline venular diameter (MU) | 146 ± 21 | 148 ± 21 | 147 ± 21 | 147 ± 21 | 147 ± 20 | 147 ± 21 | 147 ± 21 | 146 ± 21 | 0.09 |
| Flicker light-induced arteriolar dilation response (%) | 3.0 ± 2.8 | 2.7 ± 2.9 | 3.1 ± 2.8 | 3.1 ± 2.8 | 3.1 ± 2.8 | 3.0 ± 2.8 | 2.9 ± 2.8 | 3.1 ± 2.8 | 0.01 |
| Flicker light-induced venular dilation response (%) | 3.9 ± 2.2 | 3.7 ± 2.2 | 3.9 ± 2.2 | 3.9 ± 2.2 | 3.9 ± 2.2 | 3.9 ± 2.2 | 3.8 ± 2.1 | 3.9 ± 2.2 | 0.02 |
| Absolute arteriolar dilation response (delta) | 4.4 ± 3.6 | 4.0 ± 3.7 | 4.4 ± 3.6 | 4.4 ± 3.6 | 4.4 ± 3.5 | 4.4 ± 3.6 | 4.2 ± 3.5 | 4.4 ± 3.6 | 0.03 |
| Absolute venular dilation response (delta) | 7.7 ± 4.1 | 7.4 ± 4.1 | 7.7 ± 4.1 | 7.7 ± 4.1 | 7.7 ± 4.1 | 7.6 ± 4.1 | 7.4 ± 3.9 | 7.7 ± 4.1 | 0.30 |
| Skin microvascular measurements | | | | | | | | |  |
| Baseline skin blood flow before heating (PU) | 11 ± 6.6 | 11 ± 5.7 | 11 ± 6.5 | 11 ± 6.6 | 11 ± 6.6 | 11 ± 6.2 | 11 ± 6.6 | 11 ± 6.5 | 0.32 |
| Skin hyperemia during heating (PU) | 113 ± 58 | 103 ± 54 | 113 ± 58 | 113 ± 58 | 113 ± 59 | 114 ± 59 | 113 ± 58 | 113 ± 57 | 0.08 |
| Skin hyperemic response (%) | 1129 ± 773 | 1083 ± 750 | 1128 ± 770 | 1127 ± 771 | 1121 ± 781 | 1142 ± 774 | 1129 ± 773 | 1129 ± 772 | 0.33 |
| Kidney microvascular measurement | | | | | | | | | |
| Urinary albumin excretion (mg/24h) | 6.5 [4.0-12] | 7.6 [4.3-14] | 6.5 [4.0-12] | 6.5 [4.0-12] | 6.5 [3.9-12] | 6.4 [3.9-11] | 6.7 [4.1-12] | 6.5 [4.0-12] | 0.001 |

^1^ Data are presented as mean ± standard deviation for normally distributed variables, median [interquartile range] for non-normally distributed variables or percentage for categorical variables. P-value of ANOVA (for normally distributed continuous variables), Mann-Whitney U Test (for non-normally distributed continuous variables), or Chi Square test (for categorical variables) for differences between included and excluded individuals.

3-DG: 3-deoxyglucosone, CRAE: central retinal arteriolar equivalent, hsCRP: high sensitivity C-reactive protein, CRVE: central retinal venular equivalent, CVD: cardiovascular diseases, eGFR: estimated glomerular filtration rate, sE-selectin: soluble E-selectin, GO: glyoxal, sICAM-1: soluble intracellular adhesion molecule-1, IL-6: interleukin-6, IL-8: interleukin-8, MGO: methylglyoxal, NGM: normal glucose metabolism, SAA: serum amyloid A, T2DM: type 2 diabetes mellitus, TNF-α: tumor necrosis factor alpha, sVCAM-1: soluble vascular adhesion molecule-1, vWF: von Willebrand factor.

**Supplementary Table 3** – Association between dietary dicarbonyl intakes and individual plasma biomarkers of low-grade inflammation ^1^

|  | **hsCRP** | | **SAA** | **sICAM** | **IL6** | **IL-8** | **TNF-α** |
| --- | --- | --- | --- | --- | --- | --- | --- |
|  | Std. β (95% CI) | Std. β (95% CI) | | Std. β (95% CI) | Std. β (95% CI) | Std. β (95% CI) | Std. β (95% CI) |
| **Dietary MGO** | -0.05  (-0.10, -0.01) | | -0.03  (-0.08, 0.01) | 0.004  (-0.04, 0.05) | -0.02  (-0.06, 0.02) | -0.03  (-0.07, 0.02) | -0.05  (-0.10, -0.01) |
| **Dietary GO** | -0.04  (-0.09, 0.02) | | 0.002  (-0.05, 0.06) | 0.02  (-0.04, 0.07) | 0.02  (-0.03, 0.07) | -0.05  (-0.11, 0.004) | -0.01  (-0.06, 0.05) |
| **Dietary 3-DG** | -0.03  (-0.07, 0.003) | | 0.01  (-0.03, 0.05) | -0.003  (-0.04, 0.04) | -0.03  (-0.07, 0.004) | -0.03  (-0.07, 0.01) | -0.03  (-0.07, 0.01) |

^1^ All biomarkers were ln-transformed. All biomarkers and dietary dicarbonyls were standardized. Results are for fully adjusted model (model 3), adjusted for age, sex, glucose metabolism status, BMI, total energy intake, smoking status, alcohol intake, physical activity, educational level, triglycerides, systolic blood pressure, total cholesterol/HDL ratio, use of glucose-lowering-, antihypertensive- or lipid-modifying drugs.
N=2765
3-DG, 3-deoxyglucosone, hsCRP: high sensitivity C-reactive protein, GO, glyoxal, sICAM-1: soluble intracellular adhesion molecule-1, IL-6: interleukin-6, IL-8: interleukin-8, MGO, methylglyoxal, SAA: serum amyloid A, TNF-α: tumor necrosis factor alpha, sVCAM-1: soluble vascular adhesion molecule-1.

**Supplementary Table 4** - Association between dietary dicarbonyl intakes and low-grade inflammation in the Cohort on Diabetes and Atherosclerosis Maastricht (CODAM) ^1^

|  | Model | Biomarkers of low-grade inflammation  (composite score)^2^ | |
| --- | --- | --- | --- |
| Dietary MGO |  | Std. β | 95% CI |
|  | Crude | -0.06 | -0.15, 0.03 |
|  | 1 | 0.01 | -0.08, 0.09 |
|  | 2 | -0.05 | -0.16, 0.05 |
|  | 3 | -0.05 | -0.16, 0.05 |
|  |  |  |  |
| Dietary GO |  | Std. β | 95% CI |
|  | Crude | -0.07 | -0.16, 0.02 |
|  | 1 | -0.03 | -0.11, 0.06 |
|  | 2 | -0.08 | -0.21, 0.05 |
|  | 3 | -0.09 | -0.22, 0.04 |
|  |  |  |  |
| Dietary 3-DG |  | Std. β | 95% CI |
|  | Crude | -0.03 | -0.12, 0.06 |
|  | 1 | 0.01 | -0.08, 0.10 |
|  | 2 | -0.004 | -0.11, 0.10 |
|  | 3 | -0.01 | -0.11, 0.09 |

^1^ Standardized betas (β) represent 1 SD change in composite score of low-grade inflammation per 1 SD higher dietary dicarbonyl intake.
Model 1: adjusted for age + sex + glucose metabolism status
Model 2: model 1 + BMI, total energy intake, smoking status, alcohol intake, physical activity
Model 3: model 2 + triglycerides, systolic blood pressure, total cholesterol/HDL ratio, use of glucose-lowering-, antihypertensive- or lipid-modifying drugs
N=515

^2^ Composite score of low-grade inflammation consisted of the biomarkers CRP, SAA, sICAM-1, IL-6, IL-8 and TNF-α.
3-DG: 3-deoxyglucosone, GO: glyoxal, MGO: methylglyoxal

**Supplementary Table 5** - Association between dietary dicarbonyl intakes and individual plasma biomarkers of low-grade inflammation in CODAM ^1^

|  | hsCRP | SAA | sICAM | IL6 | IL-8 | TNF-α |
| --- | --- | --- | --- | --- | --- | --- |
|  | Std. β (95% CI) | Std. β (95% CI) | Std. β (95% CI) | Std. β (95% CI) | Std. β (95% CI) | Std. β (95% CI) |
| Dietary MGO | -0.08  (-0.19, 0.03) | -0.03  (-0.14, 0.08) | -0.03  (-0.14, 0.07) | -0.01  (-0.12, 0.09) | -0.03  (-0.14, 0.08) | -0.01  (-0.12, 0.11) |
| Dietary GO | -0.18  (-0.31, -0.05) | -0.04  (-0.18, 0.09) | -0.04  (-0.17, 0.08) | -0.11  (-0.24, 0.03) | 0.07  (-0.07, 0.21) | -0.02  (-0.16, 0.13) |
| Dietary 3-DG | -0.07  (-0.17, 0.04) | -0.03  (-0.14, 0.07) | 0.02  (-0.08, 0.13) | -0.02  (-0.13, 0.09) | 0.06  (-0.05, 0.17) | 0.01  (-0.11, 0.12) |

^1^ Standardized betas (β) represent 1 SD change in biomarker of low-grade inflammation per 1 SD higher dietary dicarbonyl intake. All biomarkers were ln-transformed. Results are for fully adjusted model (model 3), adjusted for age, sex, glucose metabolism status, BMI, total energy intake, smoking status, alcohol intake, physical activity, triglycerides, systolic blood pressure, total cholesterol/HDL ratio, use of glucose-lowering-, antihypertensive- or lipid-modifying drugs.
N=515
3-DG: 3-deoxyglucosone, CODAM: Cohort on Diabetes and Atherosclerosis Maastricht, hsCRP: high sensitivity C-reactive protein, GO, glyoxal, sICAM-1: soluble intracellular adhesion molecule-1, IL-6: interleukin-6, IL-8: interleukin-8, MGO, methylglyoxal, SAA: serum amyloid A, TNF-α: tumor necrosis factor alpha, sVCAM-1: soluble vascular adhesion molecule-1.

**Supplementary Table 6** – Association between dietary dicarbonyl intake and individual plasma biomarkers of endothelial function ^1^

|  | **sICAM** | **sVCAM** | **sE-selectin** | **vWF** |
| --- | --- | --- | --- | --- |
|  | Std. β (95% CI) | Std. β (95% CI) | Std. β (95% CI) | Std. β (95% CI) |
| **Dietary MGO** | 0.003 (-0.04, 0.05) | -0.02 (-0.07, 0.02) | 0.002 (-0.04, 0.05) | -0.02 (-0.06, 0.03) |
| **Dietary GO** | 0.01 (-0.04, 0.07) | 0.02 (-0.04, 0.08) | 0.01 (-0.05, 0.06) | 0.03 (-0.02, 0.09) |
| **Dietary 3-DG** | -0.01 (-0.05, 0.03) | 0.001 (-0.04, 0.04) | -0.01 (-0.05, 0.03) | -0.02 (-0.06, 0.02) |

^1^ Standardized betas (β) represent 1 SD change in biomarkers of endothelial function per 1 SD higher dietary dicarbonyl intake. sICAM and E-selectin were ln-transformed. Results are for fully adjusted model (model 3), adjusted for age, sex, glucose metabolism status, BMI, total energy intake, smoking status, alcohol intake, physical activity, educational level, triglycerides, systolic blood pressure, total cholesterol/HDL ratio, use of glucose-lowering-, antihypertensive- or lipid-modifying drugs
N=2745
3-DG, 3-deoxyglucosone, GO, glyoxal, MGO, methylglyoxal, sE-selectin, soluble e-selectin, sICAM-1: soluble intracellular adhesion molecule-1, sVCAM-1: soluble vascular adhesion molecule-1, vWF: von Willebrand factor.

**Supplementary Table 7** - Associations for which we observed an interaction stratified for glucose metabolism status ^1^

|  | Normal glucose metabolism | Prediabetes | Type 2 diabetes |
| --- | --- | --- | --- |
|  | Std. β (95% CI) | Std. β (95% CI) | Std. β (95% CI) |
|  | Retinal venular diameter | | |
| Dietary GO | -0.01 (-0.09, 0.07) | -0.03 (-0.20, 0.14) | 0.08 (-0.04, 0.21) |
|  |  | Retinal arteriolar dilation |  |
| Dietary GO | -0.06 (-0.14, 0.03) | 0.02 (-0.16, 0.20) | -0.13 (-0.26, -0.01) |
|  |  | Urinary albumin excretion |  |
| Dietary GO | 0.04 (-0.03, 0.10) | -0.07(-0.21, 0.08) | -0.13 (-0.26, -0.01) |
|  |  |  |  |
|  |  | Retinal venular diameter |  |
| Dietary 3-DG | 0.02 (-0.03, 0.08) | -0.03 (-0.18, 0.13) | -0.10 (-0.22, 0.02) |
|  |  | Retinal venular dilation |  |
| Dietary 3-DG | 0.02 (-0.03, 0.08) | -0.03 (-0.18, 0.13) | -0.10 (-0.22, 0.02) |
|  |  | Skin hyperemia ^2^ |  |
| Dietary 3-DG | -0.02 (-0.10, 0.05) | 0.13 (-0.02, 0.28) | 0.04 (-0.06, 0.14) |
|  | Urinary albumin excretion ^3^ | | |
| Dietary 3-DG | 0.01 (-0.03, 0.05) | -0.01 (-0.12, 0.11) | -0.10 (-0.21, 0.01) |

^1^ Standardized betas (β) represent 1 SD change in outcome per 1 SD higher dietary dicarbonyl intake. Results are for fully adjusted model (model 3): adjusted for age, sex, glucose metabolism status, BMI, total energy intake, smoking status, alcohol intake, physical activity, educational level, triglycerides, systolic blood pressure, total cholesterol/HDL ratio, use of glucose-lowering-, antihypertensive- or lipid-modifying drugs. Individuals with other types of diabetes were excluded in these analyses because of the small sample size (n=23). Sample sizes differ per outcome:
For retinal venular diameter: normal glucose metabolism n=1401, prediabetes n=366, type 2 diabetes n=648.
For retinal arteriolar and venular dilation: normal glucose metabolism n=1220, prediabetes n=319, type 2 diabetes n=520.
For skin hyperemia: normal glucose metabolism n=756, prediabetes n=211, type 2 diabetes n=371.
For urinary albumin excretion: normal glucose metabolism n=1604, prediabetes n=418, type 2 diabetes n=725.

^2^ Skin hyperemia was assessed as absolute average skin heating response, adjusting for baseline blood flow in all models.

^3^ Urinary albumin excretion was ln-transformed

3-DG, 3-deoxyglucosone, GO, glyoxal, MGO, methylglyoxal

**Supplementary Table 8** – Sensitivity analyses for the associations between dietary MGO and plasma biomarkers of low-grade inflammation ^1^

|  |  | Outcome: biomarkers of low-grade inflammation (composite score) ^2^ | |
| --- | --- | --- | --- |
| Dietary MGO | N | Std. β | 95% CI |
| Model 3 ^3^ | **2765** | **-0.05** | **-0.09, -0.01** |
| Kcal + DHD15 | 2765 | -0.04 | -0.08, -0.003 |
| Kcal + carbohydrates | 2765 | -0.06 | -0.10, -0.02 |
| Kcal + protein | 2765 | -0.04 | -0.08, -0.002 |
| Kcal + fat | 2765 | -0.05 | -0.10, -0.01 |
| + eGFR | 2755 | -0.04 | -0.08, -0.001 |
| + retinopathy | 2574 | -0.05 | -0.09, -0.004 |
| + urinary albumin excretion | 2748 | -0.05 | -0.09, -0.01 |
| + history of CVD | 2750 | -0.05 | -0.09, -0.01 |
| Exclusion other type of diabetes | 2738 | -0.05 | -0.09, -0.01 |
| Exclusion CRP>10 | 2646 | -0.06 | -0.11, -0.02 |
| Exclusion GI disease | 2464 | -0.04 | -0.08, 0.01 |
| Exclusion previously diagnosed diabetes | 2153 | -0.04 | -0.09, 0.01 |
| HT med. > individual HT med. | 2765 | -0.05 | -0.10, -0.01 |
| GMS > HbA1c | 2758 | -0.05 | -0.09, -0.01 |
| GMS > Fasting glucose | 2763 | -0.06 | -0.10, -0.02 |
| GMS > Post-load glucose | 2566 | -0.06 | -0.10, -0.01 |
| OSBP > 24h BP | 2395 | -0.06 | -0.10, -0.01 |
| Education > income | 2273 | -0.06 | -0.11, -0.02 |
| Education > occupational status | 2335 | -0.05 | -0.09, -0.001 |
| CHAMPS > ActivPAL | 2415 | -0.04 | -0.08, 0.004 |
| BMI > waist | 2764 | -0.04 | -0.09, -0.004 |
|  |  |  |  |

^1^ Standardized betas (β) represent 1 SD change in composite score of low-grade inflammation per 1 SD higher dietary MGO intake.

^2^ Composite score of low-grade inflammation consisted of the biomarkers CRP, SAA, sICAM-1, IL-6, IL-8 and TNF-α.

^3^ Model 3: adjusted for age, sex, glucose metabolism status, BMI, total energy intake, smoking status, alcohol intake, physical activity, educational level, triglycerides, systolic blood pressure, total cholesterol/HDL ratio, use of glucose-lowering-, antihypertensive- or lipid-modifying drugs.

BP: blood pressure, CVD: cardiovascular disease, CRP: C-reactive protein, DHD15: Dutch Healthy Diet Index 2015, eGFR: estimated glomerular filtration rate, GI: gastrointestinal, GMS: glucose metabolism status, HT med.: anti-hypertensive medication, MGO: methylglyoxal, OSBP: office systolic blood pressure.

**Supplementary table 9** - Sensitivity analyses for the associations between dietary MGO and flicker light-induced retinal venular dilation ^1^

|  |  | Outcome: Retinal venular dilation  (delta change) | |
| --- | --- | --- | --- |
| Dietary MGO | N | Std. β | 95% CI |
| Model 3 ^2^ | **2078** | **-0.07** | **-0.13, -0.01** |
| Kcal + DHD15 | 2078 | -0.07 | -0.12, -0.01 |
| Kcal + carbohydrates | 2078 | -0.07 | -0.13, -0.01 |
| Kcal + protein | 2078 | -0.06 | -0.12, -0.01 |
| Kcal + fat | 2078 | -0.07 | -0.13, -0.01 |
| + eGFR | 2062 | -0.07 | -0.12, -0.01 |
| + retinopathy | 2031 | -0.07 | -0.12, -0.01 |
| + urinary albumin excretion | 2064 | -0.07 | -0.13, -0.02 |
| + history of CVD | 2066 | -0.07 | -0.12, -0.01 |
| Exclusion other type of diabetes | 2059 | -0.06 | -0.12, -0.01 |
| Exclusion GI disease | 1850 | -0.08 | -0.13, -0.02 |
| Exclusion previously diagnosed diabetes | 1637 | -0.06 | -0.12, 0.003 |
| HT med. > individual HT med. | 2078 | -0.07 | -0.12, -0.01 |
| GMS > HbA1c (%) | 2075 | -0.07 | -0.12, -0.01 |
| GMS > Fasting glucose | 2077 | -0.07 | -0.12, -0.01 |
| GMS > Post-load glucose | 1947 | -0.07 | -0.13, -0.01 |
| OSBP > 24h BP | 1818 | -0.05 | -0.11, 0.01 |
| Education > income | 1713 | -0.07 | -0.13, -0.01 |
| Education > occupational status | 1753 | -0.10 | -0.16, -0.04 |
| CHAMPS > ActivPAL | 1848 | -0.10 | -0.16, -0.04 |
| BMI > waist | 2077 | -0.07 | -0.12, -0.01 |
|  |  |  |  |

^1^ Standardized betas (β) represent the 1 SD increase in retinal venular dilation per 1 SD higher dietary MGO intake.

^2^ Model 3: adjusted for age, sex, glucose metabolism status, BMI, total energy intake, smoking status, alcohol intake, physical activity, educational level, triglycerides, systolic blood pressure, total cholesterol/HDL ratio, use of glucose-lowering-, antihypertensive- or lipid-modifying drugs.
BP: blood pressure, CVD: cardiovascular disease, CRP: C-reactive protein, DHD15: Dutch Healthy Diet Index 2015, eGFR: estimated glomerular filtration rate, GI: gastrointestinal, GMS: glucose metabolism status, HT med.: anti-hypertensive medication, MGO: methylglyoxal, OSBP: office systolic blood pressure.

**Supplementary figures**

**
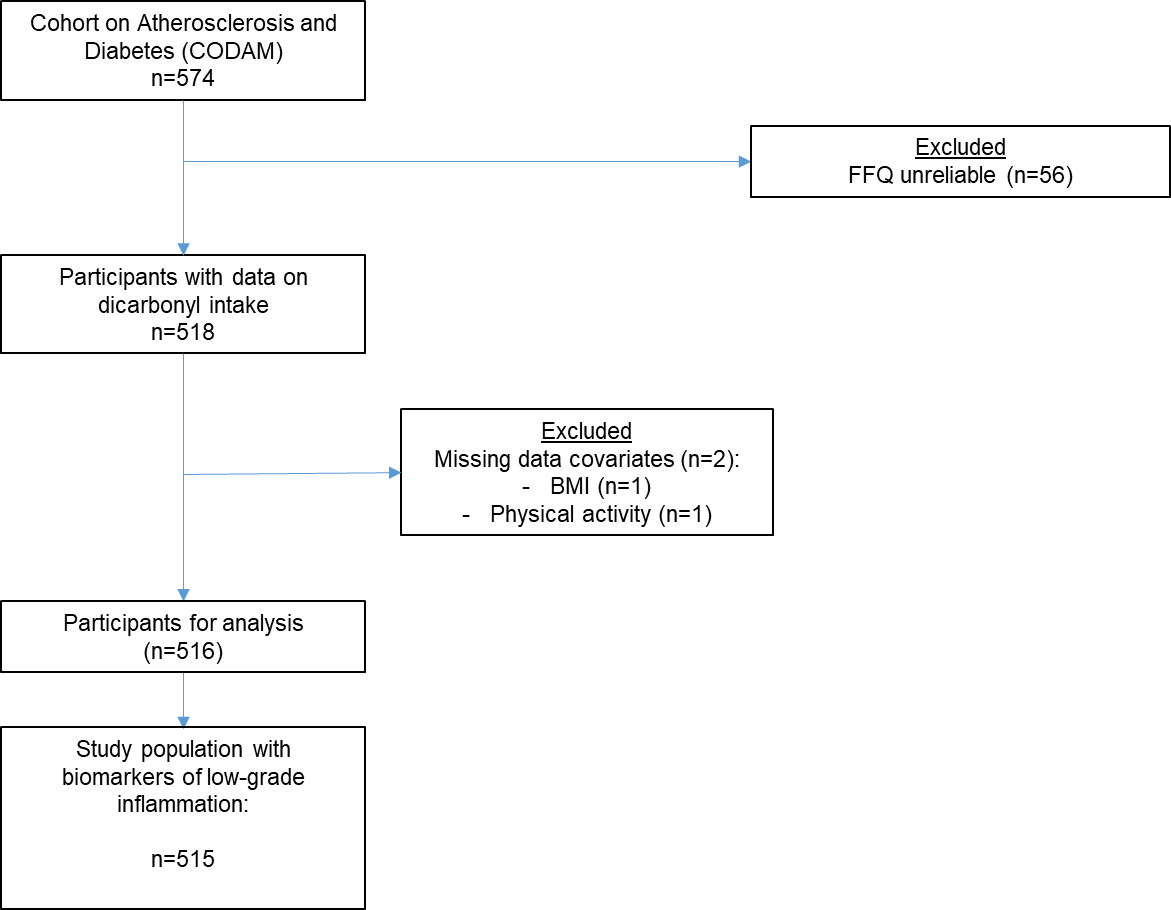
**

**Supplementary Figure 1** - Flowchart Cohort on Diabetes and Atherosclerosis Maastricht

FFQ: Food Frequency Questionnaire
